# Apathy, depression, and dementia risk in older adults: A global collaborative study

**DOI:** 10.1002/alz.71059

**Published:** 2025-12-29

**Authors:** Dae Jong Oh, Darren M. Lipnicki, Fleur Harrison, Ji Won Han, Tae Hui Kim, Kyung Phil Kwak, Bong Jo Kim, Shin Gyeom Kim, Jeong Lan Kim, Seok Woo Moon, Joon Hyuk Park, Seung‐Ho Ryu, Jong Chul Youn, Dong Young Lee, Dong Woo Lee, Seok Bum Lee, Jung Jae Lee, Jin Hyeong Jhoo, Erico Castro‐Costa, Daniel Davis, Richard B. Lipton, Mindy J. Katz, Pierre‐Marie Preux, Maëlenn Guerchet, Nikolaos Scarmeas, Mary Yannakoulia, Mary Kosmidis, Elena Rolandi, Annalisa Davin, Michele Rossi, Oye Gureje, Akin Ojagbemi, Olufisayo Elugbadebo, Steffi Riedel‐Heller, Susanne Roehr, Alexander Pabst, Henry Brodaty, Perminder S. Sachdev, Ki Woong Kim

**Affiliations:** ^1^ Workplace Mental Health Institute Kangbuk Samsung Hospital Sungkyunkwan University School of Medicine Seoul Republic of Korea; ^2^ Centre for Healthy Brain Ageing School of Clinical Medicine University of New South Wales Sydney New South Wales Australia; ^3^ Department of Neuropsychiatry Seoul National University Bundang Hospital Seongnam Republic of Korea; ^4^ Department of Psychiatry Seoul National University College of Medicine Seoul Republic of Korea; ^5^ Department of Psychiatry Yonsei University Wonju Severance Christian Hospital Wonju Republic of Korea; ^6^ Department of Psychiatry Dongguk University Gyeongju Hospital Gyeongju Republic of Korea; ^7^ Department of Psychiatry Gyeongsang National University School of Medicine Jinju Republic of Korea; ^8^ Department of Neuropsychiatry Soonchunhyang University Bucheon Hospital Bucheon Republic of Korea; ^9^ Department of Psychiatry College of Medicine Chungnam National University Jung Republic of Korea; ^10^ Department of Psychiatry and Research Institute of Medical Science School of Medicine Konkuk University Konkuk University Chungju Hospital Chungju Republic of Korea; ^11^ Department of Neuropsychiatry Jeju National University Hospital Jeju Republic of Korea; ^12^ Department of Psychiatry School of Medicine Konkuk University Konkuk University Medical Center Seoul Republic of Korea; ^13^ Department of Neuropsychiatry Kyunggi Provincial Hospital for the Elderly Yongin Republic of Korea; ^14^ Department of Neuropsychiatry Seoul National University Hospital Seoul Republic of Korea; ^15^ Department of Psychiatry Inje University Sanggye Paik Hospital Seoul Republic of Korea; ^16^ Department of Psychiatry Dankook University Hospital Cheonan Republic of Korea; ^17^ Department of Psychiatry School of Medicine Kangwon National University Chuncheon Republic of Korea; ^18^ Center for Studies in Public Health and Aging (NESPE) Belo Horizonte Brazil; ^19^ Department of Public Health & Primary Care School of Clinical Medicine University of Cambridge Cambridge UK; ^20^ Saul R. Korey Department of Neurology Albert Einstein College of Medicine of Yeshiva University Bronx New York USA; ^21^ Inserm U1094, IRD UMR270, Univ. Limoges, CHU Limoges EpiMaCT ‐ Epidemiology of Chronic Diseases in Tropical Zone Institute of Epidemiology and Tropical Neurology OmegaHealth Limoges France; ^22^ Laboratory of Epidemiology of Chronic and Neurological Diseases Faculty of Health Sciences University of Abomey‐Calavi Cotonou Benin; ^23^ 1st Department of Neurology National and Kapodistrian University of Athens Athens Greece; ^24^ Department of Neurology Columbia University New York New York USA; ^25^ Department of Nutrition and Dietetics Harokopio University Athens Greece; ^26^ Department of Cognition Brain and Behavior Aristotle University of Thessaloniki Thessaloniki Greece; ^27^ Golgi Cenci Foundation Abbiategrasso Italy; ^28^ WHO Collaborating Centre for Research and Training in Mental Health Neurosciences and Substance Abuse Department of Psychiatry University of Ibadan Ibadan Nigeria; ^29^ Institute of Social Medicine Occupational Health and Public Health (ISAP) University of Leipzig Leipzig Germany; ^30^ School of Psychology Massey University Auckland New Zealand; ^31^ Global Brain Health Institute (GBHI) Trinity College Dublin Dublin UK; ^32^ Department of Brain and Cognitive Science Seoul National University College of Natural Sciences Seoul Republic of Korea; ^33^ Institute of Human Behavioral Medicine Seoul National University Medical Research Center Seoul Republic of Korea

**Keywords:** Alzheimer's disease, apathy, cohort study, dementia, depression, mild cognitive impairment, neuropsychiatric symptoms

## Abstract

**INTRODUCTION:**

The role of apathy in predicting dementia risk, especially with coexisting depression, remains unclear in community‐dwelling older adults.

**METHODS:**

We analyzed data from 12,646 older adults without baseline dementia across ten population‐based cohorts from six continents (Cohort Studies of Memory in an International Consortium study). Apathy, depression, and dementia outcomes were assessed using harmonization protocols.

**RESULTS:**

Over 5.7 years of follow‐up, 1160 developed dementia and 552 Alzheimer's disease (AD). Apathy prevalence was 34.1% and more than three times higher in those with depression. Apathy was not associated with dementia risk among cognitively normal or non‐depressed individuals. In contrast, among individuals with depression and mild cognitive impairment (MCI), apathy predicted a higher risk of dementia (hazard ratio [HR] 1.62) and AD (HR 1.74).

**DISCUSSION:**

Apathy is associated with increased dementia risk only in older adults with MCI and depression. Findings highlight the need to incorporate affective symptom monitoring into dementia risk assessment and intervention.

**Highlights:**

- Harmonized data from 10 population‐based cohorts across six continents were used.
- Apathy predicted dementia only in older adults with both depression and mild cognitive impairment.
- No association was found in non‐depressed or cognitively normal individuals.
- Monitoring progression of affective symptoms may aid dementia risk stratification.

## 1 BACKGROUND

Dementia is a major global health concern, imposing significant burdens on patients, caregivers, and health‐care systems.^1^ Identifying early predictors of dementia is critical for implementing timely interventions and reducing its impact. Apathy, the state of diminished motivation, interest, and goal‐directed behavior,^2^ is not only one of the most common neuropsychiatric symptoms accompanying dementia, but it also frequently precedes the onset of dementia.^3^, ^4^, ^5^ Therefore, apathy may serve as a critical factor for identifying populations at risk of developing dementia.

However, the role of apathy as an independent predictor of dementia remains insufficiently understood due to limitations in prior research. Most studies on apathy and dementia have focused on individuals with mild cognitive impairment (MCI).^6^, ^7^, ^8^ The link between apathy and dementia or cognitive decline remains unclear in older adults with normal cognition. Furthermore, because most studies have been conducted in memory clinic settings, their findings may not be generalizable to community‐dwelling older adults. Although some community‐based studies have investigated the relationship between apathy and dementia, their findings have been conflicting. While some studies reported that apathy increased dementia risk,^9^, ^10^, ^11^ others found no such association.^12^, ^13^, ^14^, ^15^, ^16^ These inconsistencies may result from the lack of distinction between MCI and normal cognition, leading to variations in findings based on the extent of dementia‐related pathological burden.

A critical unresolved question is whether the association between apathy and dementia risk is moderated by depression, a well‐established risk factor for dementia.^17^ Apathy frequently presents alongside depression, and given the significant overlap between these conditions, previous studies linking apathy to dementia risk may have been largely influenced by the effects of depression. Importantly, apathy shows limited response to conventional antidepressant treatments and often requires distinct therapeutic approaches, such as behavioral interventions aimed at enhancing reward exposure.^18^ This clinical distinction further underscores the importance of examining apathy separately from depression when evaluating and managing dementia risk. A previous study analyzing MCI patients referred from Alzheimer's Disease Centers in the United States^19^ attempted to distinguish these effects, but no existing study has addressed this issue among community‐dwelling older adults. Clarifying whether apathy is independently associated with dementia risk or whether this association is moderated by depression is crucial for understanding its predictive role and guiding prevention strategies for at‐risk populations.

To address these limitations, this study aims to investigate the relationship among apathy, depression, and dementia risk using large, community‐based cohort studies from many countries with diverse and well‐defined cognitive profiles. By examining the association between apathy and dementia risk in the context of depression, this study aims to clarify both the independent and interactive effects of these affective symptoms on the risk of incident dementia. Ultimately, this study aims to enhance our understanding of how affective symptoms are associated with dementia risk, providing valuable insights for future research and intervention strategies.

## 2 METHODS

### 2.1 Study design, setting, and participants

As part of the Cohort Studies of Memory in an International Consortium (COSMIC) collaboration cohort study,^20^ 10 prospective cohorts—representing diverse ethno‐regional groups across six continents—contributed to the current analysis. These cohorts included the Bambuí Cohort Study of Aging (BCSA),^21^ Cognitive Function and Ageing Study (CFAS),^22^ Einstein Aging Study (EAS),^23^ Epidemiology of Dementia in Central Africa (EPIDEMCA),^24^ Hellenic Longitudinal Investigation of Aging and Diet (HELIAD),^25^ Invecchiamento Cerebrale in Abbiategrasso (Invece.Ab),^26^ Ibadan Study of Ageing (ISA),^27^ Korean Longitudinal Study on Cognitive Aging and Dementia (KLOSCAD),^28^ Leipzig Longitudinal Study of the Aged (LEILA75+),^29^ and Sydney Memory and Ageing Study (MAS),^30^ with a combined total of 22,198 participants. Individuals with dementia or other major psychiatric or neurological disorders, other than depression (e.g., bipolar disorder, psychotic disorders, alcohol use disorders, intellectual disability, Parkinson's disease, or epilepsy; *n* = 3038), were excluded from the study. Participants who lacked baseline information on apathy and depression (*n* = 720); key covariates such as hypertension, diabetes, or stroke (*n* = 1340); or follow‐up data (*n* = 4454) were excluded, resulting in a final analysis sample of 12,646 participants.

This study was approved by the institutional review board of the Seoul National University Bundang Hospital (IRB no. B‐0912‐089‐010) and the University of New South Wales Human Research Ethics committee (HC17292 and HC220222). All 10 contributing studies received ethics approval from their respective institutional review boards (Table S1 in supporting information), and informed consent was obtained from all participants.

### 2.2 Assessment of apathy and depression

Apathy and depression were assessed during the baseline data collection period of each cohort (Table 1). Apathy was evaluated using self‐rated questionnaires in seven studies: BCSA, EAS, HELIAD, Invece.Ab, ISA, KLOSCAD, and MAS. Among these, six studies used the 15‐item or 30‐item Geriatric Depression Scale (GDS), while the remaining one used the 12‐item General Health Questionnaire (GHQ‐12). For studies using the GDS, apathy was assessed using the GDS‐3A, which focuses on three apathy‐related items from the GDS. Apathy was diagnosed if participants endorsed at least two of these three items. The GDS‐3A is widely used as a tool for evaluating self‐rated apathy.^8^, ^31^ In contrast, the BCSA study, which used the GHQ‐12, identified apathy based on the Baltimore Epidemiologic Catchment Area study framework,^32^ in which participants were considered apathetic if they endorsed at least one of two apathy‐related items. Detailed descriptions of the specific items and evaluation methods used in each self‐reported questionnaire can be found in Table S2 in supporting information.

**RESEARCH IN CONTEXT**

1. **Systematic review**: The authors reviewed literature by searching PubMed and other databases to evaluate whether apathy predicts dementia independently in community‐dwelling older adults. While previous studies reported mixed findings—mostly limited to mild cognitive impairment (MCI) patients or clinic‐based samples, no prior research has clarified the role of coexisting depression in apathy's association with dementia.
2. **Interpretation**: Using large‐scale, population‐based cohorts from many countries, this study showed that apathy is linked to increased dementia risk only among older adults with both MCI and depression. Apathy was not associated with dementia in cognitively normal or non‐depressed MCI individuals.
3. **Future directions**: Findings suggest that depression may moderate the apathy–dementia relationship. Future research should examine how affective symptoms interact to influence dementia progression, using refined tools to distinguish apathy and depression. Biological mechanisms should be explored, and affective symptom–based risk stratification integrated into prevention alongside cognitive profiling.

**TABLE 1:** Contributing cohorts.

	BCSA	CFAS	EAS	EPIDEMCA	HELIAD	Invece.Ab	ISA	KLOSCAD	LEILA75+	MAS
Area, country	Bambuí, Brazil	Nationwide, UK	New York, USA	Central African Republic & Republic of Congo	Larissa and Marousi, Greece	Abbiategrasso, Italy	Ibadan, Nigeria	Nationwide, South Korea	Leipzig, Germany	Sydney, Australia
Data collection										
Baseline	1997	1993–1994	1993	2011–2012	2011–2014	2009–2011	2003–2004	2011–2012	1997–1998	2005–2007
Follow‐up	1998–2013	1996–2004	1994–2017	2013–2014	2014–2019	2012–2015	2007–2009	2013–2020	1999–2014	2007–2020
Interval^*^	1 year	2 years	1 year	2 years	3 years	2 years	3 years	2 years	5 years	2 years
Participants										
Non‐demented	1,326	759	1,076	554	671	638	1,186	5,010	830	596
NC^†^	N/A	N/A	865 (80.4)	517 (93.3)	604 (90.0)	590 (92.5)	N/A	3,653 (72.9)	664 (82.7)	373 (62.6)
MCI^†^	N/A	N/A	211 (19.6)	37 (6.7)	67 (10.0)	48 (7.5)	N/A	1,357 (27.1)	139 (17.3)	223 (37.4)
Ethnicity	Brazilian	White	White, African American, Hispanic	African	White	White	African	Asian	White	White
Age, years	68.8 ± 7.0	76.7 ± 6.9	77.7 ± 5.3	72.7 ± 6.3	72.7 ± 5.5	72.1 ± 1.3	74.4 ± 8.9	69.8 ± 6.6	81.6 ± 4.9	78.4 ± 4.8
Women^†^	827 (62.4)	499 (65.7)	668 (62.1)	322 (58.1)	417 (62.1)	307 (48.1)	607 (51.2)	2,832 (56.5)	601 (72.4)	356 (59.7)
Education, years	2.8 ± 3.0	9.8 ± 2.1	13.6 ± 3.5	1.6 ± 1.0	8.6 ± 5.2	6.8 ± 3.3	2.3 ± 0.9	8.3 ± 5.3	11.9 ± 1.8	11.4 ± 3.3
Hypertension^†^	824 (62.1)	81 (10.7)	674 (62.6)	377 (68.1)	471 (70.2)	424 (66.5)	118 (9.9)	2,614 (52.2)	684 (82.4)	521 (87.4)
Diabetes^†^	197 (14.9)	25 (3.3)	182 (16.9)	59 (10.6)	121 (18.0)	102 (15.5)	26 (2.2)	353 (7.0)	194 (23.4)	236 (39.6)
Stroke^†^	46 (3.5)	16 (2.1)	98 (9.1)	26 (4.7)	45 (6.7)	52 (8.2)	8 (0.7)	935 (18.7)	49 (5.9)	21 (3.5)
Follow‐up, years	10.0 ± 4.7	3.7 ± 2.3	4.5 ± 3.5	1.9 ± 0.8	3.3 ± 0.9	3.9 ± 0.6	5.4 ± 0.8	5.9 ± 2.4	5.6 ± 4.2	8.5 ± 4.2
Incident cases										
Dementia	147	86	109	28	36	32	128	257	179	158
AD	N/A	N/A	89	18	32	21	N/A	206	103	83

Abbreviations: AD, Alzheimer's disease; BCSA, Bambuí Cohort Study of Aging; CFAS, Cognitive Function and Ageing Study; EAS, Einstein Aging Study; EPIDEMCA, Epidemiology of Dementia in Central Africa; HELIAD, Hellenic Longitudinal Investigation of Aging and Diet; Invece.Ab, Invecchiamento Cerebrale in Abbiategrasso; ISA, Ibadan Study of Ageing; KLOSCAD, Korean Longitudinal Study on Cognitive Aging and Dementia; LEILA75+, Leipzig Longitudinal Study of the Aged; MAS, The Sydney Memory and Ageing Study; MCI, mild cognitive impairment; NC, normal cognition.

* Duration of interval between follow‐up assessments.

^†^ Number of participants (percentage).

Apathy was assessed through diagnostic interviews in three studies: CFAS, EPIDEMCA, and LEILA75+. Two of these studies used the General Mental State‐Automated Geriatric Examination for Computer Assisted Taxonomy (GMS‐AGECAT), while the third, LEILA75+, used the Structured Interview for the Diagnosis of Dementia of the Alzheimer Type, Multi‐infarct Dementia, and Dementias of Other Aetiology according to ICD‐10 and DSM‐III‐R (SIDAM). In the studies using the GMS‐AGECAT, four apathy‐related items—comparable to those in the GDS and GHQ‐12—were identified and scored on a scale of 0 to 2. A total score of ≥ 3 was operationally defined as apathy. In the LEILA75+ study, apathy was assessed using a single apathy‐related item in the SIDAM assessment. Further details on the evaluation items and methods are provided in Table S2.

In all studies except LEILA75+, depression was assessed using the same tools as those used for apathy evaluation. In the six studies that used the GDS, depression was defined as endorsing at least two of the remaining 11 items of the GDS‐15, excluding the three GDS‐3A items and the item directly referring to subjective memory complaints:^11^, ^33^ “Do you feel you have more problems with your memory than most people?” Similarly, for the GHQ‐12, depression was defined as endorsing at least 2 out of the remaining 10 items, excluding the apathy‐related items. In the two studies that used GMS‐AGECAT, depression was defined as having a depression score of ≥ 3.^34^ For LEILA75+, depression was assessed using the Structured Clinical Interview for the Diagnostic and Statistical Manual of Mental Disorders, 4th edition (SCID‐IV),^35^ with depression defined as meeting the criteria for major depressive disorder. Further details on the diagnostic criteria for depression in each cohort are summarized in Table S2.

### 2.3 Assessment of cognitive status and outcome variables

At baseline, MCI was identified in seven cohorts (EAS, EPIDEMCA, HELIAD, Invece.Ab, KLOSCAD, LEILA75+, and MAS), with all diagnoses made according to consensus criteria proposed by the International Working Group.^36^ Participants who did not meet the diagnostic threshold for MCI and were free from dementia were classified as having normal cognition.

The outcomes of this study were incident all‐cause dementia and incident Alzheimer's disease (AD). Outcome assessment was conducted during the follow‐up data collection period of each cohort (Table 1). All‐cause dementia was defined as an umbrella diagnosis that does not consider etiology. Due to variations in dementia definitions across the original cohorts, the diagnoses were harmonized according to the harmonization protocol provided in Table S3 in supporting information. For cases diagnosed with all‐cause dementia, seven cohorts (EAS, EPIDEMCA, HELIAD, Invece.Ab, KLOSCAD, LEILA75+, and MAS) further classified dementia subtypes based on etiology. AD was diagnosed in these cohorts using the National Institute of Neurological and Communicative Disorders and Stroke and the Alzheimer's Disease and Related Disorders Association criteria^37^ or the Diagnostic and Statistical Manual of Mental Disorders Fourth Edition (DSM‐IV)^38^ or DSM‐5 diagnostic criteria.^39^ Additional details are summarized in Table S3.

### 2.4 Assessment of covariates

Demographic factors such as age, sex, years of education, and ethnicity were included as covariates. Ethnicity was categorized as African, African American, Asian, Brazilian, Hispanic, or White. For cohorts lacking individual‐level ethnicity data, the major ethnicity of the cohort population was used as a proxy. Vascular risk factors for dementia, such as hypertension, diabetes, and stroke, are known to be related to both apathy and dementia and were therefore included as covariates to account for potential confounding, being available and harmonizable across all cohorts.^17^, ^18^, ^40^, ^41^ Hypertension was defined as the presence of systolic blood pressure ≥ 140 mmHg and/or diastolic blood pressure ≥ 90 mmHg, the use of antihypertensive medications, or a self‐reported diagnosis of hypertension by a physician. Diabetes was defined based on fasting blood glucose levels ≥ 126 mg/dL (≥ 7 mmol/L), the use of diabetes medications, or a self‐reported diagnosis of diabetes by a physician. Stroke was defined as a self‐reported diagnosis of stroke by a physician. We used all relevant data from each study to support the diagnosis or classification of the condition.

### 2.5 Statistical analysis

We estimated the crude prevalence rates (%) of apathy according to cognitive status (all non‐demented, normal cognition, and MCI) and depression status (total, non‐depressed, and depressed). The 95% confidence intervals were calculated using the Wald method.

To examine the association between apathy and depression with the risks of all‐cause dementia and AD, we conducted multivariate Cox proportional hazards analyses, adjusting for age, sex, education, ethnicity, hypertension, diabetes, stroke, and cohort. The proportional hazards assumption was verified using Schoenfeld residuals. To assess the interaction between apathy and depression, we included an apathy x depression interaction term in the proportional hazards models. All analyses were performed separately according to cognitive status (all non‐demented, normal cognition, and MCI). To ensure the robustness of our findings, we (1) conducted separate Cox proportional hazards analyses among participants from the six cohorts (EAS, HELIAD, Invece.Ab, ISA, KLOSCAD, and MAS) that used the same assessment tools for apathy and depression (GDS) and (2) performed leave‐one‐out analyses for each cohort. Additionally, a supplementary analysis was conducted using a higher threshold for self‐reported depression, in which depression was redefined as endorsing at least 4 of the 11 non‐apathy, non‐memory items on the GDS‐15 and at least 3 of the 10 non‐apathy items on the GHQ‐12.

Additionally, to determine whether the association between apathy and the risks of all‐cause dementia and AD was influenced by depression, we conducted multivariate Cox proportional hazards analyses stratified by depression status. All analyses were again performed separately by cognitive status. Furthermore, we repeated the analyses among the six cohorts using GDS and conducted leave‐one‐out analyses for each cohort.

Several sensitivity analyses were conducted to examine the robustness of the findings. (1) To minimize the potential influence of divergent observation periods and reverse causality, we performed a 2‐year landmark analysis, excluding participants with follow‐up durations of < 2 years. (2) Because the baseline hazard and observation period could differ across cohorts, we applied a stratified Cox proportional hazards model, specifying cohort as the stratification variable. (3) To account for the competing risk of death, we additionally estimated subdistribution hazard ratios using the Fine–Gray competing risk model. (4) Finally, among the cohorts for which the social interaction variable could be harmonized (BCSA, HELIAD, Invece.Ab, KLOSCAD, and LEILA75+), we further adjusted for social interaction as a covariate, as it may confound the association between apathy and dementia.^17^, ^18^

All statistical analyses were performed using the survival package in R, version 4.5.1 (R Project for Statistical Computing).

## 3 RESULTS

We included 12,646 participants without dementia at baseline (mean age: 72.6 ± 7.3 years; 58.8% women). The 10 contributing cohorts presented variations in participant numbers (ranging from 554 to 5510), study locations (Australia, Brazil, Central African Republic and Republic of Congo, Germany, Greece, Italy, Nigeria, South Korea, UK, and United States), mean age (68.8 ± 7.0 to 81.6 ± 4.9 years), proportion of women participants (48.1% to 72.4%), and mean follow‐up duration (1.9 ± 0.8 to 10.0 ± 4.7 years). A total of 1160 all‐cause dementia cases and 552 AD cases were identified over a mean follow‐up period of 5.7 ± 3.4 years. The characteristics of each cohort are summarized in Table 1.

The overall prevalence of apathy was 34.1%. Among individuals with depression, the prevalence of apathy was more than three times higher than in those without depression (56.6% vs. 18.5%). In individuals with MCI, the prevalence of apathy was 53% higher than in those with normal cognition (47.8% vs. 31.2%). Across all cognitive status groups, the presence of depression was associated with a significantly higher prevalence of apathy compared to its absence (chi‐squared test, *p* < 0.001). Notably, apathy prevalence was particularly high in individuals with MCI and comorbid depression. Table 2 presents an overview of the prevalence of apathy, while the detailed prevalence of apathy for each cohort is provided in Table S4 in supporting information.

**TABLE 2:** Crude prevalence of apathy according to depression and cognitive status.

Cognition status	Depression status	No. of cases/total	Prevalence, % (95% CI)
All non‐demented	Non‐depressed	1,390/7,498	18.5 (17.7–19.4)
	Depressed	2,916/5,148	56.6 (55.3–58.0)
	Total	4,306/12,646	34.1 (33.2–34.9)
Normal cognition	Non‐depressed	710/4,452	15.9 (14.9–17.0)
	Depressed	1,559/2,814	55.4 (53.6–57.2)
	Total	2,269/7,266	31.2 (30.2–32.3)
MCI	Non‐depressed	238/945	25.2 (22.4–28.0)
	Depressed	758/1,137	66.7 (63.9–69.4)
	Total	996/2,082	47.8 (45.7–50.0)

Abbreviations: CI, confidence interval; MCI, mild cognitive impairment.

In overall participants, apathy was associated with a 17% increased risk of all‐cause dementia, while depression was linked to a 36% higher risk. Apathy was associated with a 32% increased risk of AD, and depression with a 37% increase. In individuals with normal cognition, depression was associated with an increased risk of all‐cause dementia and AD, while apathy showed no significant associations. In contrast, among those with MCI, apathy was linked to a 26% higher risk of all‐cause dementia and a 47% higher risk of AD, with no significant associations observed for depression (see Table 3). There were similar findings when restricting the analysis to the six cohorts using the GDS. No significant interactions between apathy and depression were found regarding the risks of all‐cause dementia or AD in any analysis (Table 3). These findings were supported by results observed in leave‐one‐out analyses conducted for each cohort (see Tables S5 and S6 in supporting information), as well as in the analysis using a higher threshold for self‐reported depression (see Table S7 in supporting information).

**TABLE 3:** Association of apathy and depression with the risk of incident dementia by cognitive status.

	All‐cause dementia			Alzheimer's disease		
	No. of cases/total	HR (95% CI)	*p*	No. of cases/total	HR (95% CI)	*p*
**Total cohorts**						
All non‐demented (*n* = 12,646)						
Apathy	440/4306	**1.17 (1.01–1.34)**	**0.031**	229/3267	**1.32 (1.07–1.62)**	**0.009**
Depression	429/5148	**1.36 (1.17–1.58)**	**<0.001**	222/3951	**1.37 (1.09–1.71)**	**0.007**
Apathy x depression	–	1.14 (0.86–1.50)	0.361	–	1.22 (0.81–1.83)	0.348
Normal cognition (*n* = 7266)						
Apathy	131/2269	1.18 (0.91–1.53)	0.201	84/2269	1.10 (0.80–1.51)	0.572
Depression	118/2814	**1.36 (1.04–1.80)**	**0.028**	87/2814	**1.49 (1.06–2.08)**	**0.020**
Apathy x depression	–	1.16 (0.70–1.92)	0.563	–	1.45 (0.76–2.74)	0.257
Mild cognitive impairment (*n* = 2082)						
Apathy	175/996	**1.26 (1.00–1.60)**	**0.048**	145/996	**1.47 (1.12–1.92)**	**0.005**
Depression	166/1137	1.13 (0.86–1.51)	0.382	135/1137	0.96 (0.70–1.31)	0.783
Apathy x depression	–	1.44 (0.90–2.30)	0.129	–	1.25 (0.73–2.13)	0.418
**Cohorts using GDS**						
All non‐demented (*n* = 9177)						
Apathy	356/3607	**1.23 (1.05–1.44)**	**0.011**	222/3161	**1.37 (1.11–1.70)**	**0.004**
Depression	344/4408	**1.37 (1.15–1.64)**	**<0.001**	207/3725	**1.30 (1.03–1.65)**	**0.029**
Apathy x depression	–	1.31 (0.95–1.79)	0.099	–	1.36 (0.89–2.08)	0.160
Normal cognition (*n* = 6085)						
Apathy	123/2182	1.20 (0.92–1.58)	0.187	79/2182	1.15 (0.82–1.60)	0.433
Depression	104/2612	**1.41 (1.04–1.90)**	**0.026**	75/2612	1.38 (0.96–1.98)	0.083
Apathy x depression	–	1.40 (0.81–2.42)	0.232	–	1.89 (0.94–3.79)	0.074
Mild cognitive impairment (*n* = 1906)						
Apathy	172/979	**1.31 (1.03–1.66)**	**0.027**	143/979	**1.56 (1.18–2.06)**	**0.002**
Depression	161/1113	0.98 (0.74–1.30)	0.880	132/1113	0.92 (0.67–1.27)	0.617
Apathy x depression	–	1.45 (0.89–2.35)	0.132	–	1.24 (0.71–2.15)	0.447

Notes: The hazard ratios adjusted for age, sex, education, ethnicity, hypertension, diabetes, stroke, and cohort are presented. Statistical values with *p* < 0.05 are reported in bold.

Abbreviations: CI, confidence interval; GDS, Geriatric Depression Scale; HR, hazard ratio.

When participants were stratified by the presence of depression, apathy was not significantly associated with the risk of all‐cause dementia or AD among those without depression, both in normal cognition and MCI. In contrast, among participants with depression, apathy was associated with a 33% increased risk of all‐cause dementia and a 58% increased risk of AD. Notably, in individuals with depression and MCI, apathy was linked to a 62% higher risk of all‐cause dementia and a 74% higher risk of AD (see Table 4). These associations between apathy and dementia risk were similarly observed in analyses restricted to cohorts using the GDS; in MCI participants with GDS‐defined depression, apathy was associated with 61% and 75% higher risks of all‐cause dementia and AD, respectively. Furthermore, even in normal cognition with GDS‐defined depression, apathy was associated with a 74% increased risk of AD (Table 4). Even after adjusting for the total GDS score in cohorts using the GDS, apathy remained significantly associated with increased risks of all‐cause dementia (hazard ratio [HR] = 1.60, 95% confidence interval [CI] = 1.10–2.34, *p* = 0.014) and AD (HR = 1.83, 95% CI = 1.20–2.79, *p* = 0.005) among MCI participants with GDS‐defined depression. These findings were also confirmed in a supplementary analysis using a higher threshold for self‐reported depression (see Table S8 in supporting information). All leave‐one‐out analyses supported these findings, except when the KLOSCAD cohort was excluded, at which point apathy in MCI with GDS‐defined depression no longer showed an association with increased risks of all‐cause dementia and AD (see Table S9 in supporting information).

**TABLE 4:** Association of apathy with the risk of incident dementia by depression and cognitive status.

	All‐cause dementia			Alzheimer's disease		
	No. of cases/total of apathy	HR (95% CI) of apathy	*p*	No. of cases/total of apathy	HR (95% CI) of apathy	*p*
Non‐depressed, total cohorts						
All non‐demented	161/1390	1.05 (0.86–1.28)	0.612	72/948	1.12 (0.83–1.50)	0.463
Normal cognition	54/710	1.03 (0.73–1.45)	0.890	26/710	0.81 (0.50–1.29)	0.364
MCI	56/238	1.01 (0.72–1.41)	0.959	46/238	1.30 (0.89–1.91)	0.178
Non‐depressed, cohorts using GDS						
All non‐demented	125/1081	1.09 (0.86–1.37)	0.474	69/897	1.15 (0.85–1.56)	0.373
Normal cognition	49/665	1.00 (0.70–1.43)	0.989	24/665	0.79 (0.49–1.29)	0.350
MCI	55/232	1.10 (0.78–1.55)	0.588	45/232	1.41 (0.94–2.11)	0.093
Depressed, total cohorts						
All non‐demented	279/2916	**1.33 (1.08–1.64)**	**0.008**	157/2317	**1.58 (1.17–2.15)**	**0.003**
Normal cognition	77/1559	1.39 (0.92–2.10)	0.115	58/1559	1.56 (0.96–2.53)	0.072
MCI	119/758	**1.62 (1.14–2.31)**	**0.008**	99/758	**1.74 (1.17–2.60)**	**0.006**
Depressed, cohorts using GDS						
All non‐demented	231/2526	**1.38 (1.09–1.74)**	**0.008**	153/2264	**1.64 (1.19–2.25)**	**0.003**
Normal cognition	74/1517	1.52 (0.97–2.38)	0.068	55/1517	**1.74 (1.01–2.98)**	**0.045**
MCI	117/747	**1.61 (1.12–2.30)**	**0.010**	98/747	**1.75 (1.17–2.62)**	**0.007**

Notes: The hazard ratios adjusted for age, sex, education, ethnicity, hypertension, diabetes, stroke, and cohort are presented. Statistical values with *p* < 0.05 are reported in bold.

Abbreviations: CI, confidence interval; GDS, Geriatric Depression Scale; HR, hazard ratio; MCI, mild cognitive impairment.

Sensitivity analyses yielded results largely consistent with the main findings. In the 2‐year landmark analysis, although the hazard ratios and statistical significance were slightly attenuated, the overall pattern of associations remained essentially unchanged (Table S10 in supporting information). Similar results were obtained from the stratified Cox proportional hazards model specifying cohort as the stratification variable (Table S11 in supporting information). Comparable findings were also observed in the Fine–Gray competing risk model accounting for death as a competing event (Table S12 in supporting information). Furthermore, in the five cohorts with available data on social interaction, after harmonizing the social interaction variable (Table S13 in supporting information) and including it as an additional covariate in the Cox proportional hazards model, the associations among apathy, depression, and dementia risk remained largely unchanged (Table S14 in supporting information).

## 4 DISCUSSION

Apathy was highly prevalent in older adults, particularly among those with MCI, and was frequently comorbid with depression. This study demonstrated that apathy was associated with an increased risk of incident dementia in community‐dwelling older adults. However, no association was found between apathy and dementia risk in those without depression and/or MCI. Notably, this association was significant only in participants with coexisting depression.

In this study, apathy was associated with an increased risk of dementia only in individuals with MCI. There was no significant association in cognitively normal older adults. Our findings reveal that apathy is highly prevalent in older adults, with a rate of 31.2% in cognitively normal individuals. This may suggest that apathy can be common even in the absence of neurodegenerative diseases. These findings are in line with previous studies reporting that apathy increases with normative aging in the community.^32^, ^42^ The age‐related physiological changes, such as chronic low‐grade inflammation and inflammation‐induced cardiovascular risk factors, could cause apathy regardless of neurodegeneration via altered transmission and metabolism of dopamine and glutamate, the neurotransmitters closely linked to the reward system.^40^, ^43^, ^44^ Age‐related reduction in social networks^45^ may also be a contributing factor to diminished motivation and interest in daily life. Given these factors, assessing dementia risk based solely on the presence of apathy may be insufficient in cognitively normal older adults. Instead, it is crucial to consider contextual factors that might amplify the relationship between apathy and dementia, improving risk stratification for intervention.

Notably, apathy increased dementia risk only in the presence of coexisting depression. The co‐occurrence of apathy and depression is common among community‐dwelling older adults. In our study, 56.6% of individuals with depression and 66.7% of those with both MCI and depression also had apathy. Previous studies have linked apathy to neuronal injury and amyloid beta (Aβ) deposition in the anterior cingulate cortex and medial prefrontal cortex, whereas depression has been associated with these pathological processes in the frontal‐limbic regions, including the amygdala, hippocampus, cingulate cortex, and inferior temporal, inferior parietal, and dorsolateral prefrontal cortices.^18^, ^46^ Therefore, individuals with MCI and coexisting apathy and depression may exhibit more widespread AD‐related pathology, including amyloid burden, neuronal injury, and white matter microstructural changes that disrupt cortical–subcortical networks involved in motivation and emotion regulation.^47^ In addition to its biological underpinnings, apathetic depression is associated with greater social isolation and increased risky behaviors (e.g., sedentary behaviors and alcohol consumption),^4^, ^48^ potentially further amplifying the detrimental impact of a single affective symptom on dementia risk. Clinically, while apathy in otherwise healthy older adults may not require immediate intervention, its coexistence with depression and MCI may signal a subgroup with more advanced pathology and higher short‐term dementia risk. Such individuals may benefit from early detection and targeted interventions beyond conventional antidepressant therapy, including behavioral intervention (e.g., Enhancing Neurobehavioral Gains with the Aid of Games and Exercises [ENGAGE]), dopaminergic agents, or neuromodulation.^18^, ^49^ Our findings also highlight that monitoring the progression from apathy or depression alone to their coexistence could help identify individuals at particularly high risk for dementia, who might also represent potential candidates for amyloid biomarker assessment and future anti‐amyloid therapy trials.

Prior studies on apathy and dementia risk in community‐dwelling older adults have reported inconsistent findings, likely due to differences in participants’ cognitive status. Studies with a higher proportion of cognitively normal individuals consistently found no association between apathy and dementia risk,^12^, ^13^ whereas studies focused solely on MCI populations or studies in which baseline cognitive diagnosis was unknown showed mixed results.^9^, ^10^, ^11^, ^14^, ^15^, ^16^ These discrepancies may stem from small sample sizes, particularly the low number of individuals with apathy, which limited statistical power. Additionally, previous studies did not account for depression, a factor that modulates the apathy–dementia relationship. By differentiating the effects of cognitive status and depression, our study addresses these limitations through a large‐scale collaborative approach, allowing for greater statistical power and a more nuanced understanding of apathy as a dementia risk factor. In doing so, our findings may contribute to bridging existing knowledge gaps and offer insights into the interplay among apathy, depression, and cognitive decline.

Apathy prevalence in MCI varies widely across studies, ranging from 3.1% to 18.5%,^3^, ^10^, ^14^, ^15^, ^16^, ^50^ significantly lower than the 47.8% prevalence observed in our study. This discrepancy may be due to differences in assessment methods, as previous studies primarily used caregiver‐reported tools like the Neuropsychiatric Inventory, whereas most cohorts in our study (7 out of 10), including 6 using GDS‐3A, relied on self‐reported measures. In community‐based samples, self‐reported GDS‐3A has been associated with higher apathy prevalence, with rates of 19.8% in mixed populations^11^ and 44.8% in MCI,^51^ aligning more closely with our findings. Caregivers may underreport mild apathy, as it is often less noticeable in early stages, and reporting may vary based on caregiver–patient dynamics and cultural backgrounds.^52^, ^53^, ^54^ However, our study found that even when restricting analysis to self‐reported GDS‐3A, the association between apathy and dementia risk remained robust. This suggests that self‐reported tools may be valuable for identifying high‐risk individuals, particularly in clinical and community settings in which reliable informants are unavailable, trained examiners are not available, or a more time‐efficient assessment is needed.

This study has several limitations. First, our findings may be influenced by large sample sizes in certain cohorts. While results from leave‐one‐out analyses were generally consistent with the main analysis, statistical power was notably reduced when analyzing subgroups with fewer cases, such as depressed MCI individuals. In particular, when excluding the largest cohort (KLOSCAD), the association between apathy and dementia risk in depressed MCI lost statistical significance, and the overall trend of results shifted slightly. Therefore, although this study includes a large, multiethnic, and multinational cohort of community‐dwelling older adults, caution is needed when generalizing these results. Second, for apathy assessment, we used the GDS‐3A for most cohorts, which consists of only three items and may not fully capture the severity or broad domains of apathy. This limitation may have contributed to the lack of a significant association between apathy and dementia risk in cognitively normal individuals, as many participants classified as having apathy likely had mild severity. In addition, self‐reported assessment of apathy may have led to an underestimation of its prevalence among individuals with MCI.^55^ GDS‐3A may also have limited ability to differentiate apathy from depression, because the GDS‐3A was originally developed for assessing depressive symptoms. This potential overlap could partially explain the finding that apathy was related to dementia risk primarily among individuals with depression. To address this limitation, we attempted an analysis restricted to non‐GDS cohorts, but the limited number of apathy and dementia cases rendered the analysis infeasible. Future studies should include clinician‐rated instruments, such as the Apathy Evaluation Scale, and incorporate larger samples to better assess apathy severity and its qualitative distinctions from depression. Third, follow‐up periods varied both within and across cohorts and were relatively short (5.7 ± 3.4 years). Despite the sensitivity analyses conducted to address this issue, such heterogeneous and short observation periods may have been insufficient to fully capture dementia progression, particularly among cognitively normal individuals, and could have introduced potential bias. Fourth, despite extensive harmonization efforts across 10 international cohorts, differences in data availability and variable definitions may have influenced the analyses. In particular, definitions and ascertainment of incident dementia were not fully uniform across cohorts, and the availability of some potential covariates was limited by feasibility of harmonization.

Despite these limitations, this study provides valuable insights into the roles of apathy and depression in the progression risk of dementia in community‐dwelling older adults by using a large‐scale, multiethnic, and multinational cohort. Our results hold important implications for community‐based dementia prevention strategies, underscoring the need for early identification of high‐risk individuals and the integration of affective symptoms monitoring into routine cognitive health assessments. Future research should assess the dementia risk associated with the interplay between apathy and depression using more reliable tools. Researchers should also examine how affective symptom‐based risk stratification complements cognitive‐based classification. Furthermore, it is necessary to clarify the distinct biological mechanisms linking affective symptoms to dementia progression.

## CONFLICT OF INTEREST STATEMENT

Perminder S. Sachdev was a member of expert advisory committees of Biogen and Roche Australia in 2020‐22 and Eli Lilly in 2025. All other authors report nothing to disclose. Author disclosures are available in the supporting information.

## CONSENT STATEMENT

All participants provided written informed consent prior to inclusion in the study.

## Supporting information



Supporting information

Supporting information

## References

[1] GBD 2019 Dementia Forecasting Collaborators . Estimation of the global prevalence of dementia in 2019 and forecasted prevalence in 2050: an analysis for the Global Burden of Disease Study 2019. Lancet Public Health. 2022;7(2):e105‐e125. 34998485 10.1016/S2468-2667(21)00249-8 PMC8810394

[2] Marin RS . Apathy: a neuropsychiatric syndrome. J Neuropsychiatry Clin Neurosci. 1991;3(3):243‐254. 1821241 10.1176/jnp.3.3.243

[3] Geda YE , Roberts RO , Knopman DS , et al. Prevalence of neuropsychiatric symptoms in mild cognitive impairment and normal cognitive aging: population‐based study. Arch Gen Psychiatry. 2008;65(10):1193‐1198. 18838636 10.1001/archpsyc.65.10.1193 PMC2575648

[4] Lanctot KL , Aguera‐Ortiz L , Brodaty H , et al. Apathy associated with neurocognitive disorders: recent progress and future directions. Alzheimers Dement. 2017;13(1):84‐100. 27362291 10.1016/j.jalz.2016.05.008

[5] Lyketsos CG , Lopez O , Jones B , Fitzpatrick AL , Breitner J , DeKosky S . Prevalence of neuropsychiatric symptoms in dementia and mild cognitive impairment: results from the cardiovascular health study. JAMA. 2002;288(12):1475‐1483. 12243634 10.1001/jama.288.12.1475

[6] Fan Z , Wang L , Zhang H , et al. Apathy as a risky neuropsychiatric syndrome of progression from normal aging to mild cognitive impairment and dementia: a systematic review and meta‐analysis. Front Psychiatry. 2021;12:792168. 34987434 10.3389/fpsyt.2021.792168 PMC8721876

[7] Fresnais D , Humble MB , Bejerot S , Meehan AD , Fure B . Apathy as a predictor for conversion from mild cognitive impairment to dementia: a systematic review and meta‐analysis of longitudinal studies. J Geriatr Psychiatry Neurol. 2023;36(1):3‐17. 35446723 10.1177/08919887221093361 PMC9755689

[8] van Dalen JW , van Wanrooij LL , van Moll Charante EP , Brayne C , van Gool WA , Richard E . Association of apathy with risk of incident dementia: a systematic review and meta‐analysis. JAMA Psychiatry. 2018;75(10):1012‐1021. 30027214 10.1001/jamapsychiatry.2018.1877 PMC6233800

[9] Bock MA , Bahorik A , Brenowitz WD , Yaffe K . Apathy and risk of probable incident dementia among community‐dwelling older adults. Neurology. 2020;95(24):e3280‐e3287. 33055276 10.1212/WNL.0000000000010951 PMC7836653

[10] Pink A , Stokin GB , Bartley MM , et al. Neuropsychiatric symptoms, APOE epsilon4, and the risk of incident dementia: a population‐based study. Neurology. 2015;84(9):935‐943. 25653291 10.1212/WNL.0000000000001307 PMC4351664

[11] van Dalen JW , Van Wanrooij LL , van Moll Charante EP , Richard E , van Gool WA . Apathy is associated with incident dementia in community‐dwelling older people. Neurology. 2018;90(1):e82‐e89. 29196576 10.1212/WNL.0000000000004767 PMC5754645

[12] Acosta I , Borges G , Aguirre‐Hernandez R , Sosa AL , Prince M , Dementia Research G . Neuropsychiatric symptoms as risk factors of dementia in a Mexican population: a 10/66 dementia research group study. Alzheimers Dement. 2018;14(3):271‐279. 29028481 10.1016/j.jalz.2017.08.015 PMC5869051

[13] Brodaty H , Heffernan M , Draper B , et al. Neuropsychiatric symptoms in older people with and without cognitive impairment. J Alzheimers Dis. 2012;31(2):411‐420. 22571979 10.3233/JAD-2012-120169

[14] Peters ME , Rosenberg PB , Steinberg M , et al. Neuropsychiatric symptoms as risk factors for progression from CIND to dementia: the Cache County Study. Am J Geriatr Psychiatry. 2013;21(11):1116‐1124. 23567370 10.1016/j.jagp.2013.01.049 PMC3525756

[15] van der Linde RM , Stephan BC , Matthews FE , et al. The presence of behavioural and psychological symptoms and progression to dementia in the cognitively impaired older population. Int J Geriatr Psychiatry. 2013;28(7):700‐709. 22887592 10.1002/gps.3873

[16] Yang AN , Wang XL , Rui HR , Luo H , Pang M , Dou XM . Neuropsychiatric symptoms and risk factors in mild cognitive impairment: a cohort investigation of elderly patients. J Nutr Health Aging. 2020;24(2):237‐241.32003417 10.1007/s12603-020-1312-9

[17] Livingston G , Huntley J , Liu KY , et al. Dementia prevention, intervention, and care: 2024 report of the Lancet standing Commission. Lancet. 2024;404(10452):572‐628. 39096926 10.1016/S0140-6736(24)01296-0

[18] Steffens DC , Fahed M , Manning KJ , Wang L . The neurobiology of apathy in depression and neurocognitive impairment in older adults: a review of epidemiological, clinical, neuropsychological and biological research. Transl Psychiatry. 2022;12(1):525. 36572691 10.1038/s41398-022-02292-3 PMC9792580

[19] Ruthirakuhan M , Herrmann N , Vieira D , Gallagher D , Lanctot KL . The roles of apathy and depression in predicting alzheimer disease: a longitudinal analysis in older adults with mild cognitive impairment. Am J Geriatr Psychiatry. 2019;27(8):873‐882. 30910421 10.1016/j.jagp.2019.02.003 PMC6646066

[20] Sachdev PS , Lipnicki DM , Kochan NA , et al. COSMIC (Cohort Studies of Memory in an International Consortium): an international consortium to identify risk and protective factors and biomarkers of cognitive ageing and dementia in diverse ethnic and sociocultural groups. BMC Neurol. 2013;13:165. 24195705 10.1186/1471-2377-13-165 PMC3827845

[21] Lima‐Costa MF , Firmo JO , Uchoa E . Cohort profile: the Bambui (Brazil) Cohort Study of Ageing. Int J Epidemiol. 2011;40(4):862‐867. 20805109 10.1093/ije/dyq143

[22] Brayne C , McCracken C , Matthews FE . Medical Research Council Coginitive F, Ageing S. Cohort profile: the Medical Research Council Cognitive Function and Ageing Study (CFAS). Int J Epidemiol. 2006;35(5):1140‐1145. 16980700 10.1093/ije/dyl199

[23] Katz MJ , Lipton RB , Hall CB , et al. Age‐specific and sex‐specific prevalence and incidence of mild cognitive impairment, dementia, and Alzheimer dementia in blacks and whites: a report from the Einstein Aging Study. Alzheimer Dis Assoc Disord. 2012;26(4):335‐343. 22156756 10.1097/WAD.0b013e31823dbcfc PMC3334445

[24] Samba H , Guerchet M , Ndamba‐Bandzouzi B , et al. Dementia‐associated mortality and its predictors among older adults in sub‐Saharan Africa: results from a 2‐year follow‐up in Congo (the EPIDEMCA‐FU study). Age Ageing. 2016;45(5):681‐687. 27230914 10.1093/ageing/afw097

[25] Dardiotis E , Kosmidis MH , Yannakoulia M , Hadjigeorgiou GM , Scarmeas N . The Hellenic Longitudinal Investigation of Aging and Diet (HELIAD): rationale, study design, and cohort description. Neuroepidemiology. 2014;43(1):9‐14. 24993387 10.1159/000362723

[26] Guaita A , Colombo M , Vaccaro R , et al. Brain aging and dementia during the transition from late adulthood to old age: design and methodology of the “Invece.Ab” population‐based study. BMC Geriatr. 2013;13:98. 24063518 10.1186/1471-2318-13-98 PMC3849204

[27] Gureje O , Ogunniyi A , Kola L . The profile and impact of probable dementia in a sub‐Saharan African community: results from the Ibadan Study of Aging. J Psychosom Res. 2006;61(3):327‐333. 16938510 10.1016/j.jpsychores.2006.07.016 PMC2820716

[28] Han JW , Kim TH , Kwak KP , et al. Overview of the korean longitudinal study on cognitive aging and dementia. Psychiatry Investig. 2018;15(8):767‐774. 10.30773/pi.2018.06.02 PMC6111226 30086611

[29] Riedel‐Heller SG , Busse A , Aurich C , Matschinger H , Angermeyer MC . Prevalence of dementia according to DSM‐III‐R and ICD‐10: results of the Leipzig Longitudinal Study of the Aged (LEILA75+) Part 1. Br J Psychiatry. 2001;179:250‐254. 11532803 10.1192/bjp.179.3.250

[30] Sachdev PS , Brodaty H , Reppermund S , et al. The sydney memory and ageing study (mas): methodology and baseline medical and neuropsychiatric characteristics of an elderly epidemiological non‐demented cohort of australians aged 70‐90 years. Int Psychogeriatr. 2010;22(8):1248‐1264. 20637138 10.1017/S1041610210001067

[31] Bertens AS , Moonen JE , de Waal MW , et al. Validity of the three apathy items of the Geriatric Depression Scale (GDS‐3A) in measuring apathy in older persons. Int J Geriatr Psychiatry. 2017;32(4):421‐428. 27060966 10.1002/gps.4484

[32] Clarke DE , Ko JY , Lyketsos C , Rebok GW , Eaton WW . Apathy and cognitive and functional decline in community‐dwelling older adults: results from the Baltimore ECA longitudinal study. Int Psychogeriatr. 2010;22(5):819‐829. 20478091 10.1017/S1041610209991402 PMC2893259

[33] van der Mast RC , Vinkers DJ , Stek ML , et al. Vascular disease and apathy in old age. The Leiden 85‐Plus Study. Int J Geriatr Psychiatry. 2008;23(3):266‐271. 17621380 10.1002/gps.1872

[34] Newman SC , Sheldon CT , Bland RC . Prevalence of depression in an elderly community sample: a comparison of GMS‐AGECAT and DSM‐IV diagnostic criteria. Psychol Med. 1998;28(6):1339‐1345. 9854275 10.1017/s0033291798007442

[35] First MB , Spitzer RL , Gibbon M , Williams JBW . Structured clinical interview for DSM‐IV axis I disorders, clinician version (SCID‐CV). American Psychiatric Press; 1996.

[36] Winblad B , Palmer K , Kivipelto M , et al. Mild cognitive impairment–beyond controversies, towards a consensus: report of the international working group on mild cognitive impairment. J Intern Med. 2004;256(3):240‐246. 15324367 10.1111/j.1365-2796.2004.01380.x

[37] McKhann G , Drachman D , Folstein M , Katzman R , Price D , Stadlan EM . Clinical diagnosis of Alzheimer's disease: report of the NINCDS‐ADRDA work group under the auspices of department of health and human services task force on Alzheimer's disease. Neurology. 1984;34(7):939‐944. 6610841 10.1212/wnl.34.7.939

[38] American Psychiatric Association . Diagnostic and Statistical Manual of Mental Disorders, 4th Ed. American Psychiatric Publishing; 1994.

[39] Sachdev PS , Blacker D , Blazer DG , et al. Classifying neurocognitive disorders: the DSM‐5 approach. Nat Rev Neurol. 2014;10(11):634‐642. 25266297 10.1038/nrneurol.2014.181

[40] Ligthart SA , Richard E , Fransen NL , et al. Association of vascular factors with apathy in community‐dwelling elderly individuals. Arch Gen Psychiatry. 2012;69(6):636‐642. 22664551 10.1001/archgenpsychiatry.2011.1858

[41] Wouts L , Marijnissen RM , Oude Voshaar RC , Beekman ATF . Strengths and weaknesses of the vascular apathy hypothesis: a narrative review. Am J Geriatr Psychiatry. 2023;31(3):183‐194. 36283953 10.1016/j.jagp.2022.09.016

[42] Brodaty H , Altendorf A , Withall A , Sachdev P . Do people become more apathetic as they grow older? A longitudinal study in healthy individuals. Int Psychogeriatr. 2010;22(3):426‐436. 20003630 10.1017/S1041610209991335

[43] Eurelings LS , Richard E , Eikelenboom P , van Gool WA , Moll van Charante EP . Low‐grade inflammation differentiates between symptoms of apathy and depression in community‐dwelling older individuals. Int Psychogeriatr. 2015;27(4):639‐647. 25729001 10.1017/S1041610214002683

[44] Lucido MJ , Bekhbat M , Goldsmith DR , et al. Aiding and abetting anhedonia: impact of inflammation on the brain and pharmacological implications. Pharmacol Rev. 2021;73(3):1084‐1117. 34285088 10.1124/pharmrev.120.000043 PMC11060479

[45] Wrzus C , Hanel M , Wagner J , Neyer FJ . Social network changes and life events across the life span: a meta‐analysis. Psychol Bull. 2013;139(1):53‐80. 22642230 10.1037/a0028601

[46] Chen Y , Dang M , Zhang Z . Brain mechanisms underlying neuropsychiatric symptoms in Alzheimer's disease: a systematic review of symptom‐general and ‐specific lesion patterns. Mol Neurodegener. 2021;16(1):38. 34099005 10.1186/s13024-021-00456-1 PMC8186099

[47] Hollocks MJ , Lawrence AJ , Brookes RL , et al. Differential relationships between apathy and depression with white matter microstructural changes and functional outcomes. Brain. 2015;138(Pt 12):3803‐3815. 26490330 10.1093/brain/awv304 PMC4655344

[48] Harrison F , Mortby ME , Mather KA , Sachdev PS , Brodaty H . Apathy as a determinant of health behaviors in older adults: implications for dementia risk reduction. Alzheimers Dement (Amst). 2023;15(4):e12505. 38026759 10.1002/dad2.12505 PMC10668002

[49] Alexopoulos GS . Mechanisms and treatment of late‐life depression. Transl Psychiatry. 2019;9(1):188. 31383842 10.1038/s41398-019-0514-6 PMC6683149

[50] Onyike CU , Sheppard JM , Tschanz JT , et al. Epidemiology of apathy in older adults: the Cache County Study. Am J Geriatr Psychiatry. 2007;15(5):365‐375. 17463187 10.1097/01.JGP.0000235689.42910.0d

[51] Richard E , Schmand B , Eikelenboom P , et al. Symptoms of apathy are associated with progression from mild cognitive impairment to Alzheimer's disease in non‐depressed subjects. Dement Geriatr Cogn Disord. 2012;33(2‐3):204‐209. 22722671 10.1159/000338239

[52] Kim G , DeCoster J , Huang CH , Bryant AN . A meta‐analysis of the factor structure of the Geriatric Depression Scale (GDS): the effects of language. Int Psychogeriatr. 2013;25(1):71‐81. 22929164 10.1017/S1041610212001421

[53] Pfeifer L , Horn AB , Maercker A , Forstmeier S . Caregiver perception of apathy in persons with mild cognitive impairment or Alzheimer's disease: a longitudinal study. Aging Ment Health. 2017;21(5):494‐500. 26666575 10.1080/13607863.2015.1118678

[54] Sink KM , Covinsky KE , Barnes DE , Newcomer RJ , Yaffe K . Caregiver characteristics are associated with neuropsychiatric symptoms of dementia. J Am Geriatr Soc. 2006;54(5):796‐803. 16696746 10.1111/j.1532-5415.2006.00697.x

[55] Guercio BJ , Donovan NJ , Munro CE , et al. The apathy evaluation scale: a comparison of subject, informant, and clinician report in cognitively normal elderly and mild cognitive impairment. J Alzheimers Dis. 2015;47(2):421‐432. 26401564 10.3233/JAD-150146 PMC4602169

