# Supplementary material for: Apathy, depression, and dementia risk in older adults: A global collaborative study
Source: Alzheimers Dement. 2025 Dec 29;21(12):e71059. doi: 10.1002/alz.71059 (PMC12746046; doi:10.1002/alz.71059)
Supplement: Supplementary file 1 — Supporting information [file ALZ-21-e71059-s002.doc]

**Supplementary Table 1.** Ethics approval for studies

| **Study** | **Institutional Review Board** |
| --- | --- |
| BCSA | Ethics Boards of the Fundac¸a˜o Oswaldo Cruz in Rio de Janeiro and the Instituto Rene´ Rachou of the Fundac¸a˜o Oswaldo Cruz in Belo Horizonte, Brazil (14/2007 - CEPSH-CpqRR) |
| CFAS | Anglia and Oxford Multi-centre Research Ethics Committee (MREC) - 99/5/22; Eastern MREC – 99/5/22; Eastern MREC – 05/MREO5/37; NRES Committee East of England – 05/MRE05/37 |
| EAS | Albert Einstein College of Medicine Institutional Review Board (approval #1996-175) |
| EPIDEMCA | Ethical committees, supervised by Ministry of Public Health in Central African Republic, the CERSSA (Comité d’Ethique de la Recherche en Sciences de Santé - 00000204/DGRST/CERSAA and 00000200/MRSIT/DGRST/CERSSA) in the Republic of Congo, approved the study protocol, as well as the CPP-SOOM-IV (Comité de la Protection des Personnes Sud-Ouest Outre-Mer) in France |
| HELIAD | Institutional Ethics Review Board of the University of Thessaly (ΒΕΥ846Ψ8Ν2-32Π) |
| Invece.Ab | Ethics Committee of the University of Pavia (#3/2009) |
| ISA | UI/UCH Research Ethics Committee – UI/IRC/02/07P |
| KLOSCAD | Ethics Committee of the Seoul National University Bundang Hospital (approval #B-0912-089-010) |
| LEILA75+ | Ethics committee of the University of Leipzig (C7 79934700) |
| MAS | University of New South Wales Human Research Ethics Committee (approval #14327) |

BCSA, Bambui Cohort Study of Ageing; CFAS, Cognitive Function and Ageing Study; EAS, Einstein Aging Study; EPIDEMCA, Epidemiology of Dementia in Central Africa; HELIAD, Hellenic Longitudinal Investigation of Aging and Diet; Invece.Ab, Invecchiamento Cerebrale in Abbiategrasso; ISA, Ibadan Study of Ageing; KLOSCAD, Korean Longitudinal Study on Cognitive Aging and Dementia; LEILA75+, Leipzig Longitudinal Study of the Aged; MAS, The Sydney Memory and Ageing Study

**Supplementary Table 2. Comparison of assessment tools for** apathy and depression

| **Study** | **Apathy** | **Depression** |
| --- | --- | --- |
| BCSA | GHQ-12 (self-rated): Apathy was defined as responding "sometimes" or "never" to at least one of the following two items from the GHQ-12: (1) "Felt that you are playing a useful part in things," and (2) "Able to enjoy your normal day-to-day activities as much as usual." | GHQ-12 (self-rated): Depression was defined as responding "always" or "frequently" to a minimum of two of the remaining 10 items in the GHQ-12, with the exclusion of the two apathy-related items, or as "sometimes" or "never" for reverse-coded questions. |
| CFAS | GMS-AGECAT (diagnostic interview): Apathy was defined as a total score of ≥3 on four GMS-AGECAT items rated on a 0–2 scale: (1) "Have you had too little energy (to do the things you want to do)?", (2) "What have you enjoyed doing recently? (Has there been any change?) Did you use to enjoy doing things?", (3) "How is your interest in things? (Do you keep up your interests?)", and (4) "Do you seem to be very slowed down in your thinking recently? Worse than usual?" | GMS-AGECAT (diagnostic interview): Depression was defined as a GMS-AGECAT score of ≥3, calculated using a computerized program following a structured clinical interview. |
| EAS | GDS-15 (self-rated): Apathy was defined as a score of ≥2 on the three apathy-related items of the GDS-15 (GDS-3A); (1) "Have you dropped many of your activities and interests?" (2) "Do you prefer to stay at home rather than going out and trying new things?" (3) "Do you feel full of energy?" (reverse-coded). | GDS-15 (self-rated): Depression was defined as a score of ≥2 on the 12 depression-related items (GDS-11D) of the GDS-15, with the exclusion of the GDS-3A items and GDS item no. 10 directly referring to subjective memory complaints. |
| EPIDEMCA | GMS-AGECAT (diagnostic interview): Apathy was defined as a total score of ≥3 on four GMS-AGECAT items rated on a 0–2 scale: (1) "Have you had too little energy (to do the things you want to do)?", (2) "What have you enjoyed doing recently? (Has there been any change?) Did you use to enjoy doing things?", (3) "How is your interest in things? (Do you keep up your interests?)", and (4) "Do you seem to be very slowed down in your thinking recently? Worse than usual?" | GMS-AGECAT (diagnostic interview): Depression was defined as a GMS-AGECAT score of ≥3, calculated using a computerized program following a structured clinical interview. |
| HELIAD | GDS-15 (self-rated): Apathy was defined as a score of ≥2 on the three apathy-related items of the GDS-3A; (1) "Have you dropped many of your activities and interests?" (2) "Do you prefer to stay at home rather than going out and trying new things?" (3) "Do you feel full of energy?" (reverse-coded). | GDS-15: Depression was defined as a score of ≥2 on the GDS-11D of the GDS-15, with the exclusion of the GDS-3A items and GDS item no. 10 directly referring to subjective memory complaints. |
| Invece.Ab | GDS-15 (self-rated): Apathy was defined as a score of ≥2 on the three apathy-related items of the GDS-3A; (1) "Have you dropped many of your activities and interests?" (2) "Do you prefer to stay at home rather than going out and trying new things?" (3) "Do you feel full of energy?" (reverse-coded). | GDS-15: Depression was defined as a score of ≥2 on the GDS-11D of the GDS-15, with the exclusion of the GDS-3A items and GDS item no. 10 directly referring to subjective memory complaints. |
| ISA | GDS-30 (self-rated): Apathy was defined as a score of ≥2 on the three apathy-related items of the GDS-3A; (1) "Have you dropped many of your activities and interests?" (2) "Do you prefer to stay at home rather than going out and trying new things?" (3) "Do you feel full of energy?" (reverse-coded). | GDS-30 (self-rated): Depression was defined as a score of ≥2 on the GDS-12D of the GDS-30 (items 1, 3, 4, 7, 8, 9, 10, 15, 17, 22, and 23). |
| KLOSCAD | GDS-30 (self-rated): Apathy was defined as a score of ≥2 on the three apathy-related items of the GDS-15 (GDS-3A); (1) "Have you dropped many of your activities and interests?" (2) "Do you prefer to stay at home rather than going out and trying new things?" (3) "Do you feel full of energy?" (reverse-coded). | GDS-30 (self-rated): Depression was defined as a score of ≥2 on the GDS-12D of the GDS-30 (items 1, 3, 4, 7, 8, 9, 10, 15, 17, 22, and 23). |
| LEILA75+ | SIDAM (diagnostic interview): Apathy was defined as a "Yes" response to item 43 from the clinician's structured interview: "The subject seems to lack drive and energy, appears indifferent, apathetic, motivation is lacking." | SCID-IV (diagnostic interview): Depression was defined as a diagnosis of major depressive disorder based on DSM-IV criteria following a structured clinical interview. |
| MAS | GDS-15 (self-rated) : Apathy was defined as a score of ≥2 on the three apathy-related items of the GDS-15 (GDS-3A); (1) "Have you dropped many of your activities and interests?" (2) "Do you prefer to stay at home rather than going out and trying new things?" (3) "Do you feel full of energy?" (reverse-coded). | GDS-15: Depression was defined as a score of ≥2 on the GDS-11D of the GDS-15, with the exclusion of the GDS-3A items and GDS item no. 10 directly referring to subjective memory complaints. |

BCSA, Bambui Cohort Study of Ageing; CFAS, Cognitive Function and Ageing Study; EAS, Einstein Aging Study; EPIDEMCA, Epidemiology of Dementia in Central Africa; HELIAD, Hellenic Longitudinal Investigation of Aging and Diet; Invece.Ab, Invecchiamento Cerebrale in Abbiategrasso; ISA, Ibadan Study of Ageing; KLOSCAD, Korean Longitudinal Study on Cognitive Aging and Dementia; LEILA75+, Leipzig Longitudinal Study of the Aged; MAS, The Sydney Memory and Ageing Study; GHQ-12, 12-item General Health Questionnaire; GMS-AGECAT, General Mental State-Automated Geriatric Examination for Computer Assisted Taxonomy; GDS-15, 15-item Geriatric Depression Scale; GDS-30, 30-item Geriatric Depression Scale; SIDAM; Structured Interview for the Diagnosis of Dementia of the Alzheimer Type, Multi-infarct Dementia and Dementias of Other Aetiology according to ICD-10 and DSM-III-R; SCID-IV, Structured Clinical Interview for Diagnostic and Statistical Manual of Mental Disorders, 4th edition

**Supplementary Table 3. Definition of all-cause dementia and Alzheimer’s disease in each cohort**

| **Study** | **All-cause dementia** | **Alzheimer’s disease** |
| --- | --- | --- |
| BCSA | The Mini-Mental State Examination (MMSE) score cut-off point of 13 was determined to be suitable for Brazilian populations with low levels of formal education. | N/A |
| CFAS | Dementia was assessed using a two-stage process. The first stage involved screening interviews, including the MMSE and the Automated Geriatric Examination for Computer Assisted Taxonomy (AGECAT) to identify potential cases of dementia. In the second stage, participants were further evaluated through the Geriatric Mental State (GMS) examination which provided data for AGECAT, along with Cambridge Cognitive Examination. Dementia was defined as an AGECAT "Organicity" syndrome level of O3 or higher, indicating cognitive impairment consistent with dementia. | N/A |
| EAS | Cognitive function was assessed through a range of neuropsychological tests targeting various cognitive domains. Subjective memory impairment and functional decline were also evaluated, and, where available, informant reports on cognitive and functional impairments were included. Clinical diagnoses were made using DSM-IV criteria and were assigned during consensus case conferences involving the study neurologist and neuropsychologist. These experts conducted a comprehensive review of cognitive test results, relevant neurological signs and symptoms, and functional status evaluations. The same diagnostic procedures were applied during all follow-up assessments. To maintain consistency in diagnostic criteria over time, individuals assessed prior to the release of DSM-IV in 1994 were retrospectively reviewed and reclassified according to DSM-IV criteria. | NINCDS-ADRDA |
| EPIDEMCA | Cognitive disorders were evaluated at baseline and follow-up through a two-phase screening and diagnostic process. The Community Screening Interview for Dementia (CSI-D) was used to identify potential dementia cases, and participants scoring poorly on the CSI-D (COGSCORE ≤ 24.5) were referred for a detailed clinical evaluation by a neurologist. Neurological examinations included assessments of orientation skills, daily living activities to gauge dependence, and additional cognitive tests. A consensus diagnosis was established based on DSM-IV-TR criteria, incorporating a review of all medical records, clinical assessments, and cognitive test results. | NINCDS-ADRDA |
| HELIAD | Certified neurologists and trained neuropsychologists conducted structured interviews and performed neuropsychological evaluations. Psychometricians administered a comprehensive battery of neuropsychological tests to evaluate major cognitive domains. The data collected from all assessments were reviewed during expert consensus meetings, involving the neurologists who conducted clinical examinations and the neuropsychologists. Dementia diagnoses were made according to DSM-IV criteria, following a consistent procedure across all assessments. | NINCDS-ADRDA |
| Invece.Ab | Clinical interviews and visits are conducted by expert geriatricians, all members of the same geriatric team applying DSM-IV criteria. Each diagnostic decision is reviewed by a second physician, and in cases of disagreement, a third geriatrician, the study lead, intervenes to resolve the discrepancy. | NINCDS-ADRDA |
| ISA | A psychiatrist reviewed all available information to determine the presence or absence of dementia. This information included scores from the 10-Word Delay Recall Test and Clinician Home-based Interview to assess Function (CHIF), the interviewer’s observations of the respondent’s memory and language (documented at the end of the assessment), reported functional status (gathered from various sections of the interview and often supplemented by key informant reports), and the temporal relationship between the onset of any depressive disorder and cognitive symptoms. While the CHIF assessed higher cognitive functions through the respondent’s ability to perform instrumental ADLs, role functioning was evaluated to identify difficulties in completing both basic and instrumental ADLs. The psychiatrist integrated all these data to make a final determination regarding the presence or absence of dementia according to DSM-IV criteria. | N/A |
| KLOSCAD | Geriatric neuropsychiatrists conducted standardized diagnostic interviews and neurological examinations using the Korean version of the Consortium to Establish a Registry for Alzheimer’s Disease Assessment Packet (CERAD-K). Research neuropsychologists or trained nurses administered a comprehensive cognitive assessment, including the CERAD-K Neuropsychological Assessment Battery, Digit Span Test, Executive Clock Drawing Task, and Frontal Assessment Battery. A panel of experts finalized the diagnoses applying DSM-IV criteria to diagnose dementia. | NINCDS-ADRDA |
| LEILA75+ | At each assessment, trained physicians and psychologists conducted fully structured interviews with the study participants. A comprehensive battery of cognitive tests was administered, and additional information on cognitive and psychosocial functioning was collected through fully structured informant interviews. Consensus conferences were then held to determine dementia status based on DSM-IV criteria. | DSM-IV |
| MAS | At baseline, participants were excluded if they had a prior diagnosis of dementia, an MMSE score below 24 (adjusted for age, education, and non-English speaking background at study entry), or received a diagnosis of dementia following comprehensive baseline assessments. A detailed neuropsychological battery was administered to evaluate multiple cognitive domains. Individuals were further assessed if they scored at least 1.5 standard deviations below published normative data on both a memory and a non-memory measure, on two non-memory measures, or exhibited reduced neuropsychological scores along with a decline in activities of daily living as determined through an informant interview. Consensus diagnoses were made in accordance with DSM-IV criteria by an expert panel of neuropsychiatrists, psycho-geriatricians, and neuropsychologists, based on all available clinical, neuropsychological, laboratory, and imaging data. The same procedure was applied consistently across all assessment occasions. | DSM-5 |

BCSA, Bambui Cohort Study of Ageing; CFAS, Cognitive Function and Ageing Study; EAS, Einstein Aging Study; EPIDEMCA, Epidemiology of Dementia in Central Africa; HELIAD, Hellenic Longitudinal Investigation of Aging and Diet; Invece.Ab, Invecchiamento Cerebrale in Abbiategrasso; ISA, Ibadan Study of Ageing; KLOSCAD, Korean Longitudinal Study on Cognitive Aging and Dementia; LEILA75+, Leipzig Longitudinal Study of the Aged; MAS, The Sydney Memory and Ageing Study; DSM, Diagnostic and Statistical Manual of Mental Disorders; ADL, ‎Activities of daily living; NINCDS-ADRDA, National Institute of Neurological and Communicative Disorders and Stroke and the Alzheimer’s Disease and Related Disorders Association

**Supplementary Table 4.** Crude prevalence of apathy according to cohorts

|  | Total | | Non-depressed | | Depressed | |
| --- | --- | --- | --- | --- | --- | --- |
|  | n | % (95% CI) | n | % (95% CI) | n | % (95% CI) |
| BCSA | 563 | 42.5 (39.8 – 45.1) | 246 | 27.2 (24.3 – 30.1) | 317 | 75.3 (71.2 – 79.4) |
| CFAS | 30 | 4.0 (2.6 – 5.3) | 10 | 1.5 (0.6 – 2.4) | 20 | 21.5 (13.2 – 29.9) |
| EAS | 365 | 33.9 (31.1 – 46.8) | 209 | 25.3 (22.3 – 28.2) | 156 | 62.7 (56.6 – 68.7) |
| EPIDEMCA | 95 | 17.1 (14.0 – 20.3) | 43 | 12.8 (9.2 – 16.3) | 52 | 24.0 (18.3 – 29.6) |
| HELIAD | 94 | 14.0 (11.4 – 16.6) | 16 | 3.3 (1.7 – 4.9) | 78 | 42.4 (35.3 – 49.5) |
| Invece.Ab | 62 | 9.7 (7.4 – 12.0) | 11 | 2.5 (1.0 – 3.9) | 51 | 26.6 (20.3 – 32.8) |
| ISA | 446 | 37.6 (34.8 – 40.4) | 184 | 36.6 (32.4 – 40.8) | 262 | 38.4 (34.7 – 42.0) |
| KLOSCAD | 2,346 | 46.8 (45.4 – 48.2) | 431 | 21.6 (19.8 – 23.5) | 1,915 | 63.4 (61.7 – 65.1) |
| LEILA75+ | 11 | 1.3 (0.5 – 2.1) | 10 | 1.2 (0.5 – 2.0) | 1 | 11.1 (0.0 – 31.6) |
| MAS | 294 | 49.3 (45.3 – 53.3) | 230 | 44.7 (40.4 – 49.0) | 64 | 79.0 (70.1 – 87.9) |

CI, confidence intervals; BCSA, Bambui Cohort Study of Ageing; CFAS, Cognitive Function and Ageing Study; EAS, Einstein Aging Study; EPIDEMCA, Epidemiology of Dementia in Central Africa; HELIAD, Hellenic Longitudinal Investigation of Aging and Diet; Invece.Ab, Invecchiamento Cerebrale in Abbiategrasso; ISA, Ibadan Study of Ageing; KLOSCAD, Korean Longitudinal Study on Cognitive Aging and Dementia; LEILA75+, Leipzig Longitudinal Study of the Aged; MAS, The Sydney Memory and Ageing Study

**Supplementary Table 5. Leaving-one-out analyses for association of apathy and depression with the risk of dementia**

|  | All-cause dementia |  |  | Alzheimer’s disease |  |
| --- | --- | --- | --- | --- | --- |
|  | Apathy | Depression |  | Apathy | Depression |
|  | HR (95% CI) | HR (95% CI) |  | HR (95% CI) | HR (95% CI) |
| BCSA | 1.07 (0.93 – 1.23) | 1.42 (1.21 – 1.67)^‡^ |  | - | - |
| CFAS | 1.03 (0.90 – 1.17) | 1.27 (1.09 – 1.47)^‡^ |  | - | - |
| EAS | 0.97 (0.85 – 1.12) | 1.33 (1.14 – 1.55)^‡^ |  | 1.44 (1.14 – 1.83)^‡^ | 1.51 (1.16 – 1.97)^‡^ |
| EPIDEMCA | 1.00 (0.88 – 1.15) | 1.31 (1.13 – 1.52)^‡^ |  | 1.40 (1.13 – 1.73)^‡^ | 1.41 (1.11 – 1.79)^‡^ |
| HELIAD | 1.01 (0.88 – 1.15) | 1.31 (1.13 – 1.52)^‡^ |  | 1.35 (1.09 – 1.67)^‡^ | 1.43 (1.13 – 1.82)^‡^ |
| Invece.Ab | 1.00 (0.88 – 1.15) | 1.33 (1.14 – 1.54)^‡^ |  | 1.40 (1.13 – 1.73)^‡^ | 1.47 (1.15 – 1.86)^‡^ |
| ISA | 1.08 (0.93 – 1.25) | 1.32 (1.12 – 1.55)^‡^ |  | - | - |
| KLOSCAD | 0.95 (0.81 – 1.12) | 1.26 (1.06 – 1.49)^‡^ |  | 1.34 (1.01 – 1.77)^‡^ | 1.63 (1.21 – 2.20)^‡^ |
| LEILA75+ | 1.02 (0.89 – 1.17) | 1.33 (1.15 – 1.54)^‡^ |  | 1.33 (1.08 – 1.63)^‡^ | 1.34 (1.07 – 1.69)^‡^ |
| MAS | 1.04 (0.90 – 1.20) | 1.32 (1.13 – 1.54)^‡^ |  | 1.51 (1.20 – 1.91)^‡^ | 1.50 (1.17 – 1.94)^‡^ |

HR, hazard ratio; CI, confidence intervals; BCSA, Bambui Cohort Study of Ageing; CFAS, Cognitive Function and Ageing Study; EAS, Einstein Aging Study; EPIDEMCA, Epidemiology of Dementia in Central Africa; HELIAD, Hellenic Longitudinal Investigation of Aging and Diet; Invece.Ab, Invecchiamento Cerebrale in Abbiategrasso; ISA, Ibadan Study of Ageing; KLOSCAD, Korean Longitudinal Study on Cognitive Aging and Dementia; LEILA75+, Leipzig Longitudinal Study of the Aged; MAS, The Sydney Memory and Ageing Study

The hazard ratios adjusted for age, sex, education, ethnicity, hypertension, diabetes, stroke, and cohort are presented.

^†^*p* < 0.100; ^‡^*p* < 0.05

**Supplementary Table 6. Leaving-one-out analyses for association of apathy and depression with the risk of dementia among participants by cognitive status**

|  | All-cause dementia |  |  | Alzheimer’s disease |  |
| --- | --- | --- | --- | --- | --- |
|  | Apathy | Depression |  | Apathy | Depression |
|  | HR (95% CI) | HR (95% CI) |  | HR (95% CI) | HR (95% CI) |
| Normal cognition |  |  |  |  |  |
| EAS | 1.28 (0.96 – 1.72)^†^ | 1.57 (1.16 – 2.14)^‡^ |  | 1.27 (0.86 – 1.85) | 1.79 (1.21 – 2.65)^‡^ |
| EPIDEMCA | 1.18 (0.90 – 1.53) | 1.37 (1.02 – 1.84)^†^ |  | 1.18 (0.84 – 1.66) | 1.49 (1.04 – 2.14)^‡^ |
| HELIAD | 1.11 (0.85 – 1.45) | 1.37 (1.02 – 1.83)^‡^ |  | 1.03 (0.74 – 1.45) | 1.54 (1.08 – 2.22)^‡^ |
| Invece.Ab | 1.17 (0.90 – 1.52) | 1.43 (1.07 – 1.91)^‡^ |  | 1.12 (0.80 – 1.56) | 1.65 (1.15 – 2.36)^‡^ |
| KLOSCAD | 1.27 (0.93 – 1.73) | 1.28 (0.91 – 1.80) |  | 1.29 (0.87 – 1.93) | 1.76 (1.18 – 2.64)^‡^ |
| LEILA75+ | 1.12 (0.87 – 1.46) | 1.41 (1.06 – 1.87)^‡^ |  | 1.06 (0.77 – 1.46) | 1.47 (1.05 – 2.06)^‡^ |
| MAS | 1.22 (0.90 – 1.66) | 1.46 (1.07 – 2.00)^‡^ |  | 1.29 (0.90 – 1.86) | 1.70 (1.17 – 2.48)^‡^ |
| MCI |  |  |  |  |  |
| EAS | 1.24 (0.96 – 1.60)^†^ | 1.12 (0.81 – 1.54) |  | 1.50 (1.11 – 2.02)^‡^ | 1.05 (0.73 – 1.51) |
| EPIDEMCA | 1.24 (0.98 – 1.57)^†^ | 1.14 (0.86 – 1.52) |  | 1.52 (1.16 – 1.99)^‡^ | 1.08 (0.78 – 1.50) |
| HELIAD | 1.31 (1.03 – 1.65)^‡^ | 1.14 (0.85 – 1.53) |  | 1.58 (1.20 – 2.08)^‡^ | 1.08 (0.78 – 1.51) |
| Invece.Ab | 1.28 (1.01 – 1.62)^‡^ | 1.13 (0.85 – 1.50) |  | 1.58 (1.20 – 2.08)^‡^ | 1.05 (0.75 – 1.46) |
| KLOSCAD | 0.99 (0.71 – 1.38) | 1.19 (0.81 – 1.75) |  | 1.32 (0.89 – 1.94) | 1.23 (0.78 – 1.93) |
| LEILA75+ | 1.30 (1.03 – 1.65)^‡^ | 1.03 (0.78 – 1.37) |  | 1.53 (1.17 – 2.01)^‡^ | 0.95 (0.69 – 1.31) |
| MAS | 1.42 (1.08 – 1.86)^‡^ | 1.10 (0.81 – 1.49) |  | 1.65 (1.21 – 2.25)^‡^ | 1.04 (0.74 – 1.47) |

HR, hazard ratio; CI, confidence intervals; EAS, Einstein Aging Study; EPIDEMCA, Epidemiology of Dementia in Central Africa; HELIAD, Hellenic Longitudinal Investigation of Aging and Diet; Invece.Ab, Invecchiamento Cerebrale in Abbiategrasso; KLOSCAD, Korean Longitudinal Study on Cognitive Aging and Dementia; LEILA75+, Leipzig Longitudinal Study of the Aged; MAS, The Sydney Memory and Ageing Study; MCI, mild cognitive impairment

The hazard ratios adjusted for age, sex, education, ethnicity, hypertension, diabetes, stroke, and cohort are presented.

^†^*p* < 0.100; ^‡^*p* < 0.05

**Supplementary Table 7. Association of apathy and depression with incident dementia risk following adjustment of cutoff values for self-reported depression**

|  | All-cause dementia | | |  | Alzheimer’s disease | | |
| --- | --- | --- | --- | --- | --- | --- | --- |
|  | No. of cases/total | HR (95% CI) | *p* |  | No. of cases/total | HR (95% CI) | *p* |
| Total cohorts |  |  |  |  |  |  |  |
| All non-demented (n = 12,646) |  |  |  |  |  |  |  |
| Apathy | 440/4,306 | 1.14 (0.99 – 1.31) | 0.072 |  | 229/3,267 | **1.28 (1.04 – 1.58)** | **0.020** |
| Depression | 330/3,369 | **1.46 (1.25 – 1.71)** | **<0.001** |  | 154/2,307 | **1.57 (1.23 – 2.00)** | **<0.001** |
| Apathy * depression | - | 0.99 (0.74 – 1.32) | 0.942 |  | - | 0.99 (0.74 – 1.32) | 0.942 |
| Normal cognition (n = 7,266) |  |  |  |  |  |  |  |
| Apathy | 131/2,269 | 1.18 (0.92 – 1.53) | 0.200 |  | 84/2,269 | 1.07 (0.78 – 1.48) | 0.673 |
| Depression | 73/1,551 | **1.45 (1.06 – 1.98)** | **0.020** |  | 57/1,551 | **1.73 (1.19 – 2.51)** | **0.004** |
| Apathy * depression | - | 0.98 (0.55 – 1.77) | 0.958 |  | - | 1.04 (0.52 – 2.08) | 0.906 |
| Mild cognitive impairment (n = 2,082) |  |  |  |  |  |  |  |
| Apathy | 175/996 | 1.26 (0.99 – 1.59) | 0.055 |  | 145/996 | **1.45 (1.11 – 1.91)** | **0.007** |
| Depression | 119/756 | 1.15 (0.86 – 1.54) | 0.341 |  | 97/756 | 1.00 (0.73 – 1.39) | 0.987 |
| Apathy * depression | - | 1.39 (0.82 – 2.37) | 0.225 |  | - | 1.24 (0.67 – 2.27) | 0.493 |
| Cohorts using GDS |  |  |  |  |  |  |  |
| All non-demented (n = 9,177) |  |  |  |  |  |  |  |
| Apathy | 356/3,607 | **1.21 (1.03 – 1.42)** | **0.024** |  | 222/3,161 | **1.33 (1.08 – 1.65)** | **0.009** |
| Depression | 214/2,396 | **1.49 (1.23 – 1.80)** | **<0.001** |  | 139/2,078 | **1.51 (1.17 – 1.94)** | **0.002** |
| Apathy * depression | - | 1.42 (0.97 – 2.08) | 0.071 |  | - | 1.33 (0.80 – 2.20) | 0.272 |
| Normal cognition (n = 6,085) |  |  |  |  |  |  |  |
| Apathy | 123/2,182 | 1.21 (0.92 – 1.59) | 0.175 |  | 79/2,182 | 1.11 (0.79 – 1.56) | 0.548 |
| Depression | 59/1,349 | **1.49 (1.05 – 2.10)** | **0.025** |  | 45/1,349 | **1.65 (1.09 – 2.49)** | **0.018** |
| Apathy * depression | - | 1.23 (0.60 – 2.53) | 0.569 |  | - | 1.44 (0.61 – 3.37) | 0.407 |
| Mild cognitive impairment (n = 1,906) |  |  |  |  |  |  |  |
| Apathy | 172/979 | **1.28 (1.00 – 1.63)** | **0.047** |  | 143/979 | **1.54 (1.17 – 2.04)** | **0.002** |
| Depression | 114/732 | 1.08 (0.80 – 1.45) | 0.619 |  | 94/732 | 0.97 (0.70 – 1.34) | 0.839 |
| Apathy * depression | - | 1.39 (0.80 – 2.43) | 0.245 |  | - | 1.24 (0.66 – 2.33) | 0.509 |

HR, hazard ratio; CI, confidence interval; MCI, mild cognitive impairment; GDS, Geriatric Depression Scale; GHQ, General Health Questionnaire

The hazard ratios adjusted for age, sex, education, ethnicity, hypertension, diabetes, stroke, and cohort are presented. In the studies using the GDS, depression was defined as endorsing ≥4 of 11 non-apathy, non-memory items; for the GHQ-12, as endorsing ≥3 of 10 non-apathy items.

**Supplementary Table 8. Association of apathy with the risk of incident dementia by depression and cognitive status following adjustment of cutoff values for self-reported depression**

|  | All-cause dementia | | |  | Alzheimer’s disease | | |
| --- | --- | --- | --- | --- | --- | --- | --- |
|  | No. of cases  /total of apathy | HR (95% CI)  of apathy | *p* |  | No. of cases  /total of apathy | HR (95% CI)  of apathy | *p* |
| Non-depressed, total cohorts |  |  |  |  |  |  |  |
| All non-demented | 212/2,142 | 1.14 (0.96 – 1.36) | 0.137 |  | 110/1,720 | 1.19 (0.93 – 1.52) | 0.174 |
| Normal cognition | 80/1,290 | 1.18 (0.88 – 1.58) | 0.266 |  | 44/1,290 | 1.00 (0.69 – 1.45) | 0.992 |
| MCI | 80/428 | 1.08 (0.81 – 1.43) | 0.621 |  | 66/428 | 1.36 (0.98 – 1.89) | 0.065 |
| Non-depressed, cohorts using GDS |  |  |  |  |  |  |  |
| All non-demented | 186/1,954 | 1.12 (0.93 – 1.36) | 0.243 |  | 107/1,667 | 1.22 (0.95 – 1.56) | 0.127 |
| Normal cognition | 75/1,245 | 1.17 (0.87 – 1.59) | 0.298 |  | 42/1,245 | 1.00 (0.68 – 1.47) | 0.994 |
| MCI | 79/422 | 1.15 (0.86 – 1.55) | 0.341 |  | 65/422 | **1.46 (1.04 – 2.05)** | **0.028** |
| Depressed, total cohorts |  |  |  |  |  |  |  |
| All non-demented | 228/2,164 | 1.19 (0.93 – 1.54) | 0.167 |  | 119/1,547 | **1.60 (1.06 – 2.42)** | **0.024** |
| Normal cognition | 51/979 | 1.19 (0.66 – 2.13) | 0.561 |  | 40/979 | 1.32 (0.69 – 2.54) | 0.405 |
| MCI | 95/568 | **1.76 (1.09 – 2.83)** | **0.021** |  | 79/568 | **1.92 (1.12 – 3.29)** | **0.018** |
| Depressed, cohorts using GDS |  |  |  |  |  |  |  |
| All non-demented | 170/1,653 | **1.47 (1.05 – 2.07)** | **0.026** |  | 115/1,494 | **1.68 (1.08 – 2.64)** | **0.023** |
| Normal cognition | 48/937 | 1.33 (0.67 – 2.65) | 0.421 |  | 37/937 | 1.52 (0.68 – 3.37) | 0.308 |
| MCI | 93/557 | **1.74 (1.07 – 2.83)** | **0.025** |  | 78/557 | **1.96 (1.13 – 3.41)** | **0.017** |

HR, hazard ratio; CI, confidence interval; MCI, mild cognitive impairment; GDS, Geriatric Depression Scale; GHQ, General Health Questionnaire

The hazard ratios adjusted for age, sex, education, ethnicity, hypertension, diabetes, stroke, and cohort are presented. In the studies using the GDS, depression was defined as endorsing ≥4 of 11 non-apathy, non-memory items; for the GHQ-12, as endorsing ≥3 of 10 non-apathy items.

**Supplementary Table 9. Leaving-one-out analyses for association between apathy and the risk of dementia among depressed participants**

|  | All-cause dementia |  | Alzheimer’s disease |
| --- | --- | --- | --- |
|  | HR (95% CI) |  | HR (95% CI) |
| All non-demented |  |  |  |
| EAS | 1.11 (0.90 – 1.37)^‡^ |  | 1.59 (1.15 – 2.18)^‡^ |
| EPIDEMCA | 1.16 (0.95 – 1.43) |  | 1.61 (1.17 – 2.20)^‡^ |
| HELIAD | 1.11 (0.90 – 1.36) |  | 1.52 (1.11 – 2.07)^‡^ |
| Invece.Ab | 1.11 (0.91 – 1.37) |  | 1.55 (1.14 – 2.11)^‡^ |
| KLOSCAD | 0.95 (0.72 – 1.24) |  | 1.27 (0.76 – 2.12) |
| LEILA75+ | 1.12 (0.92 – 1.37) |  | 1.54 (1.14 – 2.08)^‡^ |
| MAS | 1.11 (0.91 – 1.36) |  | 1.53 (1.13 – 2.07)^‡^ |
| Normal cognition |  |  |  |
| EAS | 1.43 (0.94 – 2.18)^†^ |  | 1.63 (0.98 – 2.71)^†^ |
| EPIDEMCA | 1.52 (0.98 – 2.35)^†^ |  | 1.77 (1.05 – 2.99)^‡^ |
| HELIAD | 1.16 (0.76 – 1.76) |  | 1.31 (0.79 – 2.15) |
| Invece.Ab | 1.33 (0.88 – 2.02) |  | 1.47 (0.90 – 2.40) |
| KLOSCAD | 1.41 (0.76 – 2.59) |  | 1.53 (0.78 – 2.98) |
| LEILA75+ | 1.29 (0.86 – 1.93) |  | 1.46 (0.90 – 2.34) |
| MAS | 1.31 (0.87 – 1.98) |  | 1.50 (0.93 – 2.42)^†^ |
| Mild cognitive impairment |  |  |  |
| EAS | 1.49 (1.03 – 2.15)^‡^ |  | 1.53 (1.01 – 2.30)^‡^ |
| EPIDEMCA | 1.46 (1.03 – 2.06)^‡^ |  | 1.59 (1.08 – 2.36)^‡^ |
| HELIAD | 1.68 (1.17 – 2.42)^‡^ |  | 1.81 (1.20 – 2.72)^‡^ |
| Invece.Ab | 1.49 (1.05 – 2.12)^‡^ |  | 1.66 (1.11 – 2.47)^‡^ |
| KLOSCAD | 0.81 (0.40 – 1.63) |  | 0.95 (0.43 – 2.10) |
| LEILA75+ | 1.52 (1.07 – 2.16)^‡^ |  | 1.62 (1.10 – 2.40)^‡^ |
| MAS | 1.51 (1.06 – 2.14)^‡^ |  | 1.65 (1.11 – 2.44)^‡^ |

HR, hazard ratio; CI, confidence intervals; EAS, Einstein Aging Study; EPIDEMCA, Epidemiology of Dementia in Central Africa; HELIAD, Hellenic Longitudinal Investigation of Aging and Diet; Invece.Ab, Invecchiamento Cerebrale in Abbiategrasso; KLOSCAD, Korean Longitudinal Study on Cognitive Aging and Dementia; LEILA75+, Leipzig Longitudinal Study of the Aged; MAS, The Sydney Memory and Ageing Study; MCI, mild cognitive impairment

The hazard ratios adjusted for age, sex, education, ethnicity, hypertension, diabetes, stroke, and cohort are presented.

^†^*p* < 0.100; ^‡^*p* < 0.05

**Supplementary Table 10. Association of apathy with the risk of incident dementia by depression and cognitive status in 2-year landmark analyses**

|  | All-cause dementia | |  | Alzheimer’s disease | |
| --- | --- | --- | --- | --- | --- |
|  | HR (95% CI) | *p* |  | HR (95% CI) | *p* |
| Total cohorts |  |  |  |  |  |
| All non-demented | 1.10 (0.95 – 1.28) | 0.202 |  | 1.22 (0.98 – 1.53) | 0.082 |
| Normal cognition | 1.14 (0.86 – 1.50) | 0.361 |  | 1.09 (0.77 – 1.53) | 0.626 |
| MCI | 1.14 (0.88 – 1.47) | 0.314 |  | 1.33 (0.99 – 1.78) | 0.056 |
| Cohorts using GDS |  |  |  |  |  |
| All non-demented | 1.18 (1.00 – 1.39) | 0.055 |  | **1.26 (1.01 – 1.58)** | **0.043** |
| Normal cognition | 1.18 (0.89 – 1.56) | 0.240 |  | 1.13 (0.80 – 1.60) | 0.486 |
| MCI | 1.20 (0.93 – 1.56) | 0.166 |  | **1.40 (1.04 – 1.90)** | **0.026** |
| Non-depressed, total cohorts |  |  |  |  |  |
| All non-demented | 0.99 (0.81 – 1.22) | 0.934 |  | 1.00 (0.72 – 1.38) | 0.993 |
| Normal cognition | 0.95 (0.66 – 1.36) | 0.767 |  | 0.80 (0.49 – 1.31) | 0.379 |
| MCI | 0.95 (0.66 – 1.37) | 0.784 |  | 1.16 (0.75 – 1.79) | 0.500 |
| Non-depressed, cohorts using GDS |  |  |  |  |  |
| All non-demented | 1.05 (0.83 – 1.34) | 0.668 |  | 1.05 (0.76 – 1.46) | 0.770 |
| Normal cognition | 1.00 (0.70 – 1.45) | 0.982 |  | 0.82 (0.50 – 1.34) | 0.423 |
| MCI | 1.04 (0.71 – 1.52) | 0.844 |  | 1.27 (0.81 – 2.00) | 0.294 |
| Depressed, total cohorts |  |  |  |  |  |
| All non-demented | 1.23 (0.98 – 1.53) | 0.073 |  | **1.48 (1.06 – 2.06)** | **0.020** |
| Normal cognition | 1.44 (0.91 – 2.26) | 0.120 |  | 1.59 (0.93 – 2.73) | 0.090 |
| MCI | 1.42 (0.97 – 2.07) | 0.072 |  | **1.55 (1.01 – 2.36)** | **0.043** |
| Depressed, cohorts using GDS |  |  |  |  |  |
| All non-demented | **1.29 (1.02 – 1.65)** | **0.037** |  | **1.50 (1.08 – 2.09)** | **0.016** |
| Normal cognition | 1.47 (0.93 – 2.33) | 0.099 |  | 1.66 (0.96 – 2.86) | 0.070 |
| MCI | 1.42 (0.97 – 2.07) | 0.072 |  | **1.55 (1.01 – 2.36)** | **0.043** |

HR, hazard ratio; CI, confidence interval; MCI, mild cognitive impairment; GDS, Geriatric Depression Scale

The hazard ratios adjusted for age, sex, education, ethnicity, hypertension, diabetes, stroke, and cohort are presented.

**Supplementary Table 11. Association of apathy with the risk of incident dementia by depression and cognitive status in the Cox proportional hazard stratified by the term of cohort**

|  | All-cause dementia | |  | Alzheimer’s disease | |
| --- | --- | --- | --- | --- | --- |
|  | HR (95% CI) | *p* |  | HR (95% CI) | *p* |
| Total cohorts |  |  |  |  |  |
| All non-demented | **1.18 (1.02 – 1.36)** | **0.023** |  | **1.32 (1.08 – 1.63)** | **0.008** |
| Normal cognition | 1.18 (0.91 – 1.52) | 0.217 |  | 1.10 (0.80 – 1.51) | 0.573 |
| MCI | **1.25 (1.00 – 1.59)** | **0.047** |  | **1.48 (1.13 – 1.94)** | **0.005** |
| Cohorts using GDS |  |  |  |  |  |
| All non-demented | **1.23 (1.05 – 1.44)** | **0.012** |  | **1.35 (1.09 – 1.68)** | **0.005** |
| Normal cognition | 1.18 (0.90 – 1.55) | 0.227 |  | 1.13 (0.81 – 1.58) | 0.478 |
| MCI | **1.27 (1.00 – 1.63)** | **0.039** |  | **1.50 (1.14 – 1.97)** | **0.004** |
| Non-depressed, total cohorts |  |  |  |  |  |
| All non-demented | 1.05 (0.86 – 1.28) | 0.612 |  | 1.12 (0.83 – 1.50) | 0.463 |
| Normal cognition | 1.03 (0.73 – 1.45) | 0.890 |  | 0.81 (0.50 – 1.29) | 0.364 |
| MCI | 1.01 (0.72 – 1.41) | 0.959 |  | 1.30 (0.89 – 1.91) | 0.178 |
| Non-depressed, cohorts using GDS |  |  |  |  |  |
| All non-demented | 1.09 (0.86 – 1.37) | 0.474 |  | 1.15 (0.85 – 1.56) | 0.373 |
| Normal cognition | 1.00 (0.70 – 1.43) | 0.989 |  | 0.79 (0.49 – 1.29) | 0.350 |
| MCI | 1.10 (0.78 – 1.55) | 0.588 |  | 1.41 (0.94 – 2.11) | 0.093 |
| Depressed, total cohorts |  |  |  |  |  |
| All non-demented | **1.30 (1.06 – 1.61)** | **0.014** |  | **1.52 (1.12 – 2.06)** | **0.007** |
| Normal cognition | 1.28 (0.85 – 1.93) | 0.236 |  | 1.48 (0.91 – 2.40) | 0.114 |
| MCI | **1.56 (1.09 – 2.22)** | **0.015** |  | **1.69 (1.14 – 2.53)** | **0.010** |
| Depressed, cohorts using GDS |  |  |  |  |  |
| All non-demented | **1.33 (1.05 – 1.68)** | **0.016** |  | **1.56 (1.13 – 2.15)** | **0.006** |
| Normal cognition | 1.38 (0.89 – 2.16) | 0.154 |  | 1.64 (0.95 – 2.80) | 0.074 |
| MCI | **1.54 (1.08 – 2.21)** | **0.018** |  | **1.75 (1.17 – 2.62)** | **0.007** |

HR, hazard ratio; CI, confidence interval; MCI, mild cognitive impairment; GDS, Geriatric Depression Scale

The hazard ratios adjusted for age, sex, education, ethnicity, hypertension, diabetes, and stroke are presented.

**Supplementary Table 12. Association of apathy with the risk of incident dementia by depression and cognitive status in the Fine-Gray competing risk models**

|  | All-cause dementia | |  | Alzheimer’s disease | |
| --- | --- | --- | --- | --- | --- |
|  | sHR (95% CI) | *p* |  | sHR (95% CI) | *p* |
| Total cohorts |  |  |  |  |  |
| All non-demented | 1.12 (0.97 – 1.28) | 0.122 |  | 1.21 (0.99 – 1.47) | 0.058 |
| Normal cognition | 1.12 (0.87 – 1.43) | 0.389 |  | 1.03 (0.76 – 1.41) | 0.830 |
| MCI | 1.10 (0.89 – 1.37) | 0.370 |  | **1.31 (1.02 – 1.70)** | **0.035** |
| Cohorts using GDS |  |  |  |  |  |
| All non-demented | 1.15 (0.99 – 1.35) | 0.066 |  | **1.25 (1.02 – 1.53)** | **0.032** |
| Normal cognition | 1.12 (0.87 – 1.45) | 0.384 |  | 1.06 (0.77 – 1.46) | 0.709 |
| MCI | 1.11 (0.89 – 1.39) | 0.351 |  | **1.32 (1.02 – 1.71)** | **0.035** |
| Non-depressed, total cohorts |  |  |  |  |  |
| All non-demented | 0.97 (0.80 – 1.18) | 0.758 |  | 0.97 (0.72 – 1.30) | 0.840 |
| Normal cognition | 0.91 (0.65 – 1.27) | 0.573 |  | 0.70 (0.44 – 1.12) | 0.139 |
| MCI | 0.94 (0.70 – 1.28) | 0.702 |  | 1.20 (0.84 – 1.72) | 0.309 |
| Non-depressed, cohorts using GDS |  |  |  |  |  |
| All non-demented | 0.97 (0.78 – 1.21) | 0.791 |  | 0.99 (0.73 – 1.33) | 0.921 |
| Normal cognition | 0.85 (0.60 – 1.20) | 0.359 |  | 0.66 (0.41 – 1.08) | 0.101 |
| MCI | 0.95 (0.69 – 1.29) | 0.728 |  | 1.20 (0.83 – 1.73) | 0.338 |
| Depressed, total cohorts |  |  |  |  |  |
| All non-demented | **1.32 (1.07 – 1.63)** | **0.011** |  | **1.51 (1.12 – 2.02)** | **0.006** |
| Normal cognition | 1.42 (0.95 – 2.12) | 0.087 |  | **1.61 (1.00 – 2.58)** | **0.049** |
| MCI | 1.35 (0.96 – 1.88) | 0.081 |  | **1.48 (1.02 – 2.15)** | **0.040** |
| Depressed, cohorts using GDS |  |  |  |  |  |
| All non-demented | **1.36 (1.08 – 1.71)** | **0.010** |  | **1.56 (1.14 – 2.13)** | **0.005** |
| Normal cognition | 1.55 (0.99 – 2.42) | 0.054 |  | **1.80 (1.05 – 3.09)** | **0.033** |
| MCI | 1.33 (0.95 – 1.87) | 0.092 |  | **1.49 (1.02 – 2.17)** | **0.039** |

sHR, subdistribution hazard ratio; CI, confidence interval; MCI, mild cognitive impairment; GDS, Geriatric Depression Scale

The hazard ratios adjusted for age, sex, education, ethnicity, hypertension, diabetes, stroke, and cohort are presented.

**Supplementary Table 13. Definition of social interaction with friends or family in each cohort**

| **Study** | **Harmonization: Never = 0; Few times a year = 1; Few times a month = 2; One or more times per week = 3** |  |  |
| --- | --- | --- | --- |
| BCSA | ‘In the past month, how often have you visited or received visits from your children who do not live with you?’ or ‘Not considering your children, have you visited or received regular visits from relatives in the last month who do not live in the same house as you?’ or ‘How often do you find someone from your job social gatherings outside of work hours? (excludes lunches or snacks with coworkers on weekdays)’ or ‘Do you have friends, acquaintances or neighbors that visit you or that you visit? (not necessarily the same person each time)?’: Never or almost never = 0, less than once a month/once or twice a year: 1, one to three times a month = 2, daily/one to four times a week = 3 |  |  |
| CFAS | N/A |  |  |
| EAS | N/A |  |  |
| EPIDEMCA | N/A |  |  |
| HELIAD | Out to visit friends or relatives: once a year or rarer = 0, many times a year (< once a month) = 1, many times a month (1-5 times a month) = 2, many times a week/everyday or almost everyday = 3 |  |  |
| Invece.Ab | ‘Contact with neighbors in the last month’ or ‘contact with son in the last month’ or ‘contact with daughter in the last month’ or ‘contact with sister in the last month’ or ‘contact with brother in the last month’ or ‘contact with friends in the last month’ or ‘contact with other relatives in the last month’: not present = 0, never or less than one time a month = 1, sometimes, one to three times a month = 2, often, four or more times a month = 3 |  |  |
| ISA | N/A |  |  |
| KLOSCAD | Social interaction with family or peers (counts/month): 0 = 0, present but less than once = 1, 1-4 =2, 4 or more = 3 |  |  |
| LEILA75+ | ‘How often do you see any of your children or other relatives to speak to?’ or ‘If you have friends in this community/neighbourhood, how often do you have a chat or do something with one of your friends?’ or ‘How often do you see any of your neighbours to have a chat with or do something with one of your friends?’: never/no relatives = 0, less often = 1, at least monthly = 2, daily/2-3 times a weekly/at least weekly = 3 |  |  |
| MAS | N/A |  |  |

BCSA, Bambui Cohort Study of Ageing; CFAS, Cognitive Function and Ageing Study; EAS, Einstein Aging Study; EPIDEMCA, Epidemiology of Dementia in Central Africa; HELIAD, Hellenic Longitudinal Investigation of Aging and Diet; Invece.Ab, Invecchiamento Cerebrale in Abbiategrasso; ISA, Ibadan Study of Ageing; KLOSCAD, Korean Longitudinal Study on Cognitive Aging and Dementia; LEILA75+, Leipzig Longitudinal Study of the Aged; MAS, The Sydney Memory and Ageing Study; N/A, not available

**Supplementary Table 14. Association of apathy with risk of incident dementia by depression and cognitive status in cohorts with available social interaction variables**

|  | All-cause dementia | |  | Alzheimer’s disease | |
| --- | --- | --- | --- | --- | --- |
|  | HR (95% CI) | *p* |  | HR (95% CI) | *p* |
| Total cohorts |  |  |  |  |  |
| All non-demented | 1.17 (1.01 – 1.34) | 0.031 |  | **1.32 (1.07 – 1.62)** | **0.009** |
| Normal cognition | 1.18 (0.91 – 1.53) | 0.201 |  | 1.10 (0.80 – 1.51) | 0.572 |
| MCI | 1.23 (0.98 – 1.55) | 0.078 |  | **1.47 (1.12 – 1.92)** | **0.005** |
| Cohorts using GDS |  |  |  |  |  |
| All non-demented | **1.23 (1.05 – 1.44)** | **0.011** |  | **1.37 (1.11 – 1.70)** | **0.004** |
| Normal cognition | 1.20 (0.92 – 1.58) | 0.187 |  | 1.15 (0.82 – 1.60) | 0.433 |
| MCI | **1.31 (1.03 – 1.66)** | **0.027** |  | **1.56 (1.18 – 2.06)** | **0.002** |
| Non-depressed, total cohorts |  |  |  |  |  |
| All non-demented | 1.05 (0.86 – 1.28) | 0.612 |  | 1.12 (0.83 – 1.50) | 0.463 |
| Normal cognition | 1.03 (0.73 – 1.45) | 0.890 |  | 0.81 (0.50 – 1.29) | 0.364 |
| MCI | 1.01 (0.72 – 1.41) | 0.959 |  | 1.30 (0.89 – 1.91) | 0.178 |
| Non-depressed, cohorts using GDS |  |  |  |  |  |
| All non-demented | 1.09 (0.86 – 1.37) | 0.474 |  | 1.15 (0.85 – 1.56) | 0.373 |
| Normal cognition | 1.00 (0.70 – 1.43) | 0.989 |  | 0.79 (0.49 – 1.29) | 0.350 |
| MCI | 1.10 (0.78 – 1.55) | 0.588 |  | 1.41 (0.94 – 2.11) | 0.093 |
| Depressed, total cohorts |  |  |  |  |  |
| All non-demented | **1.33 (1.08 – 1.64)** | **0.008** |  | **1.58 (1.17 – 2.15)** | **0.003** |
| Normal cognition | 1.39 (0.92 – 2.10) | 0.115 |  | 1.56 (0.96 – 2.53) | 0.072 |
| MCI | **1.62 (1.14 – 2.31)** | **0.008** |  | **1.74 (1.17 – 2.60)** | **0.006** |
| Depressed, cohorts using GDS |  |  |  |  |  |
| All non-demented | **1.38 (1.09 – 1.74)** | **0.008** |  | **1.64 (1.19 – 2.25)** | **0.003** |
| Normal cognition | 1.52 (0.97 – 2.38) | 0.068 |  | **1.74 (1.01 – 2.98)** | **0.045** |
| MCI | **1.61 (1.12 – 2.30)** | **0.010** |  | **1.75 (1.17 – 2.62)** | **0.007** |

HR, hazard ratio; CI, confidence interval; MCI, mild cognitive impairment; GDS, Geriatric Depression Scale

The hazard ratios adjusted for age, sex, education, ethnicity, hypertension, diabetes, stroke, cohort, and social interaction are presented.
